# Sex differences in the tumor promoting effects of tobacco smoke in a cRaf transgenic lung cancer disease model

**DOI:** 10.1007/s00204-023-03671-5

**Published:** 2024-01-21

**Authors:** Shen Zhong, Jürgen Borlak

**Affiliations:** Centre for Pharmacology and Toxicology, Hannover Medical School, Carl-Neuberg-Str. 1, 30625 Hannover, Germany

**Keywords:** Lung cancer, cRaf, Tobacco smoke, Tumor growth, Circadian rhythm, Sex hormone receptors

## Abstract

Tobacco smoke (TS) is the leading cause for lung cancer (LC), and female smokers are at a greater risk for LC. Yet, the underlying causes are unknown. We performed whole genome scans in TS exposed wild type and histologically characterized tumor lesions of cRaf transgenic mice. We constructed miRNA-gene and transcription factor-miRNA/gene regulatory networks and determined sex-specific gene regulations by evaluating hormone receptor activities. We validated the findings from TS exposed cRaf mice in a large cohort of smoking and never-smoking LC patients. When compared to males, TS prompted a sevenfold increase in tumor multiplicity in cRaf females. Genome-wide scans of tumor lesions identified 161 and 53 genes and miRNAs, which code for EGFR/MAPK signaling, cell proliferation, oncomirs and oncogenes, and 50% of DEGs code for immune response and tumor evasion. Outstandingly, in transgenic males, TS elicited upregulation of 20 tumor suppressors, some of which are the targets of the androgen and estrogen receptor. Conversely, in females, 18 tumor suppressors were downregulated, and five were specifically repressed by the estrogen receptor. We found TS to perturb the circadian clock in a sex-specific manner and identified a female-specific regulatory loop that consisted of the estrogen receptor, miR-22-3p and circadian genes to support LC growth. Finally, we confirmed sex-dependent tumor promoting effects of TS in a large cohort of LC patients. Our study highlights the sex-dependent genomic responses to TS and the interplay of circadian clock genes and hormone receptors in the regulation of oncogenes and oncomirs in LC growth.

**Supplementary Information:**

The online version contains supplementary material available at 10.1007/s00204-023-03671-5.

## Background

Lung cancer (LC) is the leading cause of cancer-related mortality worldwide (Siegel et al. 2023), and cigarette smoking is the primary reason for it (Hecht 2012; IARC Working Group on the Evaluation of Carcinogenic Risks to Humans 2004). So far, more than 7000 chemicals have been identified in cigarette smoke, and of the many harmful chemicals, at least 70 cause cancer (Centers for Disease Control and Prevention, (US) et al. 2010). Remarkably, female smokers are at a higher risk of developing LC (Stapelfeld et al. 2020), and such sex disparities may arise from deficient DNA repair, i.e., females have more DNA adducts per pack-year when compared to males while the expression of CYP1A1 is increased among female LC patients (Mollerup et al. 1999). Importantly, the CYP1A1 monooxygenase catalyzes the production of reactive metabolites which bind to DNA and, therefore, propagate DNA adduct formation. Indeed, a correlation exists between CYP1A1 tumor tissue expression and DNA adduct levels among LC patients (Mollerup et al. 1999). Furthermore, female sex hormones are more potent in inhibiting enzymes which detoxify nicotine-derived nitrosamine ketone, i.e., a carcinogen that originates from tobacco smoke exposures (Stapelfeld and Maser 2017).

Recently, we reported sex-disparities in LC growth in lung tumors among female cRaf transgenic mice. In the present study, we examined the tumor promoting effects of tobacco smoke in this transgenic disease model and identified complex regulatory gene networks consisting of miRNAs, hormone receptors and target genes. Our study enabled us to address the question why tobacco smoke exposure accelerated tumor growth especially among females. To corroborate findings, we performed cancer genomics in a large cohort of LC patients and investigated sex-related differences in the tumor genomes of female and male smokers. Eventually, we identified genes mechanistically linked to tumor growth and confirmed their relevance by correlating their expression with size of human lung tumors and determined the prognostic value of highly regulated genes and miRNAs.

Overall, our study provided new insight into the molecular pathogenesis of LC growth following cRaf overexpression. We report the complex interplay of the cRaf kinase/MAPK signaling pathway and the regulation of tumor suppressors, oncogenes and oncomirs by sex hormone receptors following tobacco smoke exposure.

## Methods

### SPC cRaf transgenic mice

We performed the study in accordance with the American Association for Laboratory Animal Science Policy on the Human Care and Use of Laboratory Animals. Approval to carry out animal studies was granted by the ethical review board of the Lower Saxony State Office for Customer Protection and Food Safety (LAVES), Germany (Az: 33-42502-04/869 and 33-42502-06/1081). The original cRaf transgenic mouse model stems from the laboratory of Prof. Ulf Rapp (University of Würzburg, Germany), and targeted overexpression of cRaf induces tumor growth (Kerkhoff et al. 2000). The cRaf transgenics lack the regulatory NH2-terminal sequences of the cRaf protein and, therefore, is constitutively active without interaction with upstream regulators such as RAS. By employing the gene specific promoter of the surfactant protein SP-C the cRaf-truncated protein is specifically expressed in respiratory epithelium of the lung (SP-C/c-Raf transgenic). However, unlike the original animal model that was bred in a C57/BL6 and 2 DBA hybrid background, we kept the transgenic mouse line in a C57/BL6 background.

The lung tissue samples of tobacco smoke exposed animals were donations from Walter Stinn, Philip Morris Research Laboratories GmbH, Cologne, Germany and were obtained during a Good Laboratory Practice compliant study that was conducted at the Fraunhofer Institute of Toxicology and Experimental Medicine (Fh-ITEM), Hannover, Germany, as detailed below.

### Tobacco smoke exposure

The mice were bred at the Fh-ITEM, Hannover, Germany and at the age of 5 months transported to the Philip Morris Research Laboratories at Leuven, Belgium. Prior to their assignment to the various study arms, the animals were permitted to acclimatize for 2 weeks.

Mice were placed inside whole-body exposure chambers (type WBEC85), and the sham animals were exposed to a continuous flow of filtered, conditioned fresh air, whereas the tobacco smoke groups were exposed to the reference cigarette 2R4F (Kentucky Reference Cigarette). The concentrations of total particle matter, carbon monoxide, nicotine and aldehydes were monitored simultaneously. The exposure started between 07:30 and 09:00 a.m. and consisted of a 6 h/day, 5 days/week cycle for 3 months. Supplementary Table S1 provides a synoptic view of the different study groups and after careful organ procurement, the lungs were shock frozen and stored at -80℃ until further processing.

### Histopathology

Lung tissues were fixed in 4% buffered formaldehyde in PBS for approximately 20 h, dehydrated and embedded in paraffin (Roti-Plast™, Roth, Karlsruhe, Germany). Tissue sections were obtained with a microtome and stained with hematoxylin and eosin according to standard protocols. The Gomori stain was performed according to an SOP.

### Whole genome miRNA profiling

We performed whole genome miRNA profiling of lung tissue of cRaf transgenic mice as described before (Vescovo et al. 2013, Zhong and Borlak 2023). We used the following protocol for the Affymetrix platform: from each lung we isolated 200 ng of total RNA and labeled nucleic acids with the FlashTag Biotin HSR labeling kit according to the manufacturer’s instructions (Genisphere, Hatfield, PA, USA, http://media.affymetrix.com/support/downloads/manuals/mirna_flashtag_manual.pdf). We hybridized the samples onto the Affymetrix GeneChip® miRNA array 1.0, which contains 722 and 690 mouse mature and pre-miRNAs, respectively. All experimental procedures followed the manufacturer's protocol.

In the case of the Agilent platform, we dephosphorylated 100 ng of total RNA and performed 3ʹ end labeling with the Cy3-pCp dye, purified the samples with Micro Bio-Spin columns and hybridized the samples onto arrays with the miRNA Microarray System labeling kit V2 according to the manufacturer’s instructions (https://www.agilent.com/store/en_US/Prod-5190-0456/5190-0456). The Agilent mouse miRNA microarray (Release 12.0, catalogue ID G4472B) contains 612 mouse mature miRNAs (https://www.agilent.com/cs/library/usermanuals/public/G4170-90011.pdf). We scanned the hybridized microarray slides with an Agilent DNA Microarray Scanner G2505C and analyzed the data with the Agilent ScanControl version 8.1.3 software. We processed the scanned TIFF images numerically, applied QC tools and corrected for background and outlier pixels with the Agilent Feature Extraction Software version 10.7.7.1.

### Whole genome gene expression profiling

We performed whole genome gene expression profiling of lung tissue of cRaf transgenic mice as reported previously (Rohrbeck et al. 2009; Rohrbeck and Borlak 2009). We prepared independent pools of four mice/pool, thus totaling 16 animals per study group and isolated the RNA of the mice lung with the miRNeasy Mini Kit (QIAGEN, Germany) according to the manufacturer’s instruction. Next, we checked the RNA quantity, purity and integrity of the 18S and 28S ribosomal bands by capillary electrophoresis with the Agilent 2100 Bioanalyzer system and the NanoDrop ND-1000. We used 8 µg of RNA as starting material to prepare cDNA with the GeneChip® one-cycle cDNA Kit (Affymetrix) and achieved the clean-up of double-stranded cDNA with the GeneChip® Sample Cleanup module (Affymetrix).

We used 12 µl of cDNA solution for the in vitro transcription assay (GeneChip® IVT Labeling Kit, Affymetrix) and purified the reaction product with the GeneChip® Sample Cleanup module (Affymetrix) according to manufacturer's recommendation. We quantified the purified cRNA and checked the quality with the Agilent 2100 Bioanalyzer system and the NanoDrop ND-1000. We prepared cleaved cRNA by metal-induced hydrolysis and determined the degree of fragmentation and the size of the fragmented biotinylated cRNA by capillary electrophoresis. Typically, we obtained fragments of the size of 35–200 bases.

We hybridized 10 µg of biotinylated fragmented cRNA to the GeneChip® Mouse Genome 430 2.0 array. The hybridization was set to 16 h at 60 rpm and 45 °C in a GeneChip® Hybridization Oven 640 (Affymetrix) followed by a washing and staining step of the arrays in the GeneChip® Fluidics Station 400 (Affymetrix). We performed an antibody signal amplification with streptavidin R-phycoerythrin, followed by a washing and staining protocol (Affymetrix) (SAPE; Invitrogen, USA). To amplify signals, we added the SAPE solution twice with a biotinylated anti-streptavidin antibody (Vector Laboratories, CA) and a staining step in between.

We scanned the arrays on a GeneChip® Scanner 3000 and visually inspected scanned images for artifacts. We scaled each image to the same target value for comparison between chips. We used the GeneChip® Operating Software to control the fluidics station and the scanner, to capture probe array data and to analyze hybridization intensity data. Finally, we applied default parameters of the Affymetrix software package for analysis.

### Reverse transcription quantitative real-time PCR (RT-qPCR)

We performed RT-qPCR assays with the Roche LightCycler system as described previously (Maaser and Borlak 2008). A summary of the experimental conditions and details of the gene sequences is given in Supplementary Table S2. We confirmed specificity of primers by agarose-gel electrophoresis of PCR products and calculated differences in gene expression by the 2-(ΔΔ−CT)-method with peptidylprolyl isomerase B as housekeeping gene. The data are fold changes relative to sham exposed animals.

### Data processing and statistical analysis

#### Differentially expressed genes (DEGs)

To delineate the effects of tobacco smoke and the combined effect, i.e., the interaction between cRaf and tobacco smoke, we performed various comparisons as summarized in Supplementary Fig. S1. We applied the unpaired t-test to compare the average signal values between different treatment conditions of animals. DEGs with a false discovery rate (FDR) < 0.05 and a fold change (FC) ≥|2| were considered statistically significant.

#### Differentially expressed miRNA (DEMs)

Raw signal intensity data of the Agilent and Affymetrix microarrays were uploaded onto the geneXplain platform and normalized with the LIMMA and the Robust Multi-array Average algorithm. We performed the principal component analysis to identify animals who grossly differed in their genomic responses. We used the hypergeometric test to calculate statistical significance of DEMs. For each miRNA, we calculated FC and standard deviation by comparing the signal intensity of each sample in the treatment group to the average signal intensity of the controls (sham animals). We considered miRNAs with a corrected FDR < 0.05 and FC ≥|2| as statistically significant.

We compiled the DEGs and DEMs in Supplementary Table S3.

### Gene ontology (GO) enrichment analysis and immune cell marker identification

We searched for enriched GO terms for DEGs using Metascape software (https://metascape.org/) (Zhou et al. 2019) and considered significantly enriched terms based on the criteria *p*-value < 0.05 (Supplementary Table S4). We visualized the results with ggplot2 package in R (R Core Team 2020; Wickham 2016).

We queried the CellMarker database (http://biocc.hrbmu.edu.cn/CellMarker/index.jsp) and searched literature to identify immune cell markers (Supplementary Table S5).

### MiRNA-gene regulatory networks

We searched for experimentally validated miRNA target genes by querying the miRNet 2.0 database (https://www.mirnet.ca/miRNet/home.xhtml) (Chang et al. 2020a). We compared DEGs identified in the present study to database entries of miRNet 2.0 and constructed miRNA-gene regulatory networks with Cytoscape 3.9.1 (Otasek et al. 2019).

### Transcription factor analysis

We searched for potential transcription factor binding sites (TFBSs) in DEGs using the TRANSFAC® database. Promoter regions were defined as sequences from − 2000 to + 100 bp relative to the transcription start sites. TFBSs in the promoters of DEGs with a fold enrichment ratio ≥ 1.5 and adj.*p*-value < 0.05 were considered to be statistically significant. We interrogated GSEA (https://www.gsea-msigdb.org/gsea/msigdb/genesets.jsp?collection=TFT), Transmir v2.0 database (https://www.cuilab.cn/transmir) and hTFtarget database (http://bioinfo.life.hust.edu.cn/hTFtarget#!/) to identify ChIP-seq validated TF target genes and miRNAs. We only considered DEGs and DEMs with proven experimental evidence for the actually binding of TF proteins to recognition sites in promoters of regulated genes.

### Translational research

We downloaded miRNA and mRNA sequencing data as well as clinical data of the TCGA lung adenocarcinoma (LUAD) dataset from the Xena database (Goldman et al. 2020). To explore the effect of tobacco smoke, we included 211 female smokers, 54 female non-smokers, 208 male smokers and 20 male non-smokers. LC patients without tobacco smoking history data were removed from the analysis. DEGs and DEMs were identified using Deseq2 package in R (R Core Team 2020; Love et al. 2014). We considered genes and miRNAs with |FC|> 2, FDR corrected *p*-value < 0.05 as significantly regulated. To identify genes and miRNAs linked to tumor size, we included 266 female and 224 male LC patients with tumor size data. We performed the Wilcoxon test to determine the difference between groups, *p* < 0.05 was considered statistically significant. For survival analysis, we included 491 LC patients with overall survival (OS) information. To delineate prognostic value of sex-specific genes/miRNAs, we considered 265 females and 226 male LC patients. We divided the patients into high and low expression groups according to the median value of the gene/miRNA expression, and constructed Kaplan–Meier curves to determine OS and performed univariate COX proportional hazards regression analysis determine hazard ratio.

### Statistics

We used an online tool of the institutional animal care and use committee of Boston University to calculate the sample size (https://www.bu.edu/research/ethics-compliance/animal-subjects/animal-care/research/sample-size-calculations-iacuc/). Based on the assumption of a two-fold difference between sexes, the power analysis suggested a sample size of at least four animals at an alpha level 0.05. We prepared independent pools of four mice/pool, thus totaling 16 animals per study group and used 8 animals to identify sex-dependent differences in tumor burden based on histopathology. Equally, for the miRNA expression studies, we used 6 animals per sex.

## Results

We considered sham and tobacco smoke exposed wild type (WT) and cRaf transgenic mice, and performed whole genome scans to identify DEGs and DEMs (Supplementary Fig. S1). First, we determined the effects of tobacco smoke on the pulmonary genome of WT mice by comparing tobacco smoke to sham exposed animals. Second, we compared the pulmonary genomes of tobacco smoke exposed transgenic animals to cRaf sham exposed controls. Third, we probed for the combined effect by comparing WT sham to tobacco smoke exposed transgenic animals and searched for sex-specific responses. A summary of the various study arms and experimental groups is given in Supplementary Table S1. Furthermore, to construct TF-miRNA-gene regulatory networks, we considered experimentally validated miRNA-gene targets, and searched for TF binding sites in the promoters of DEGs and DEMs. Finally, we performed translational research by evaluating a large cohort of human lung adenocarcinoma cases and show the work flow in Supplementary Fig. S2.

### Histopathology of the lung of tobacco smoke exposed cRaf transgenic mice

We previously reported the pathology of cRaf transgenic tumors, and its progression from epithelial dysplasia to LC (Rohrbeck et al. 2009; Rohrbeck and Borlak 2009). Essentially, targeted overexpression of cRaf caused distinct morphological changes of the respiratory epithelium, i.e., atypical adenomatous hyperplasia, multifocal tumor growth and eventually adenocarcinomas which consumed the entire lobe in animals with end-stage disease. Importantly, the tumor growth is not triggered by mutational events of common oncogenes or tumor suppressors, i.e., Lmyc1, p53, Tslc1 and Kras as evidenced by DNA sequencing (Supplementary file S1).

**Fig. 1:**
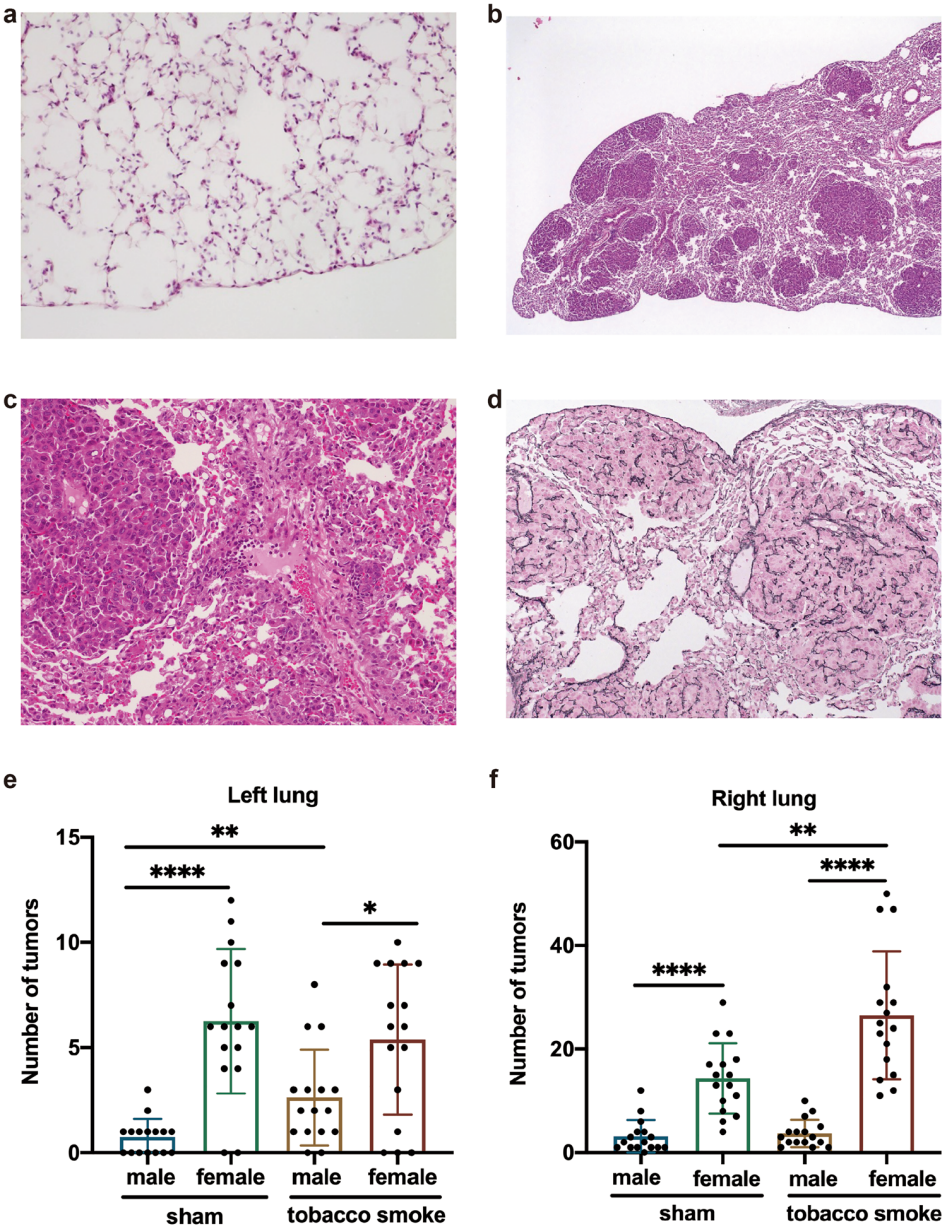
Histopathology of the lung and tumor multiplicity of tobacco smoke exposed cRaf transgenic mice. **a** H&E stain of a lung section of cRaf transgenic mice without tumor growth aged 4 month. Note the minimal thickened alveolar septa. **b** H&E stain highlighting multifocal tumor growth aged 8 month. **c** Gomori stain. Shown in black are the reticular fiber networks and in pink the desmoplastic growth pattern. **d** H&E stain highlighting infiltrating histiocytes at the rim of a tumor. **e**, **f** Multiplicity of tumors sized > 200 µm in the left and right lung of sham and tobacco smoke exposed transgenic mice. *p < 0.05, **p < 0.01, ****p < 0.0001, two-tailed Mann–Whitney *U* test

Depicted in Fig. 1a is a non-tumors lung section of a cRaf transgenic animal with regular alveolar septa and pneumocytes. Given the transgenic nature and targeted expression of cRaf in alveolar epithelium, the multifocal tumor growth is not unexpected (Fig. 1b). The foci have identical cellular features yet they differ in size which is suggestive for sequential offspring. Figure 1c depicts cellular infiltrates, i.e., monocyte-derived histiocytes and macrophages at the rim of a tumor foci and tremendously enhanced desquamation of tumor cells with activated alveolar macrophages filling the residual alveolar lumina between tumor foci. We employed the Gomori stain (Fig. 1d) to show thickened recticulin fiber network in contrast to the faint reticulin network of regular alveolar septa (black stain). Clearly visible are desmoplastic tumor reactions besides destructive growth in the various lung tumor foci.

To determine the effects of tobacco smoke exposure on lung tumor growth, we performed serial sectioning of lung tissue and counted the number of tumors sized > 200 µm. Depicted in Fig. 1e are tumor counts for the left lung of sham exposed animals. When compared to males, the tumor multiplicity was significantly increased in transgenic females (*p* < 0.01). Although tobacco smoke exposure did not significantly influence tumor multiplicity in the left lung of transgenic females (Fig. 1e), the tumor counts increased significantly (*p* < 0.01) in the right lung of transgenic females (Fig. 1f). Note, the left lung consists of a single lobe whereas the right lung consists of four lobes (superior, middle, inferior and post-caval lobe), and therefore, the difference in size likely accounted for the differences between the two lung wings. Irrespective of the anatomical location and exposure of mice to tobacco smoke, we always observed an increase in tumor multiplicity in female animals. Collectively, we obtained strong evidence for the tumor promoting effects of tobacco smoke which was significant in terms of tumor multiplicity. Furthermore, our findings underscore the importance of the cRaf/MAPK signaling pathway in LC (Witschi 2007).

### Genomic responses to tobacco smoke exposure

**Fig. 2:**
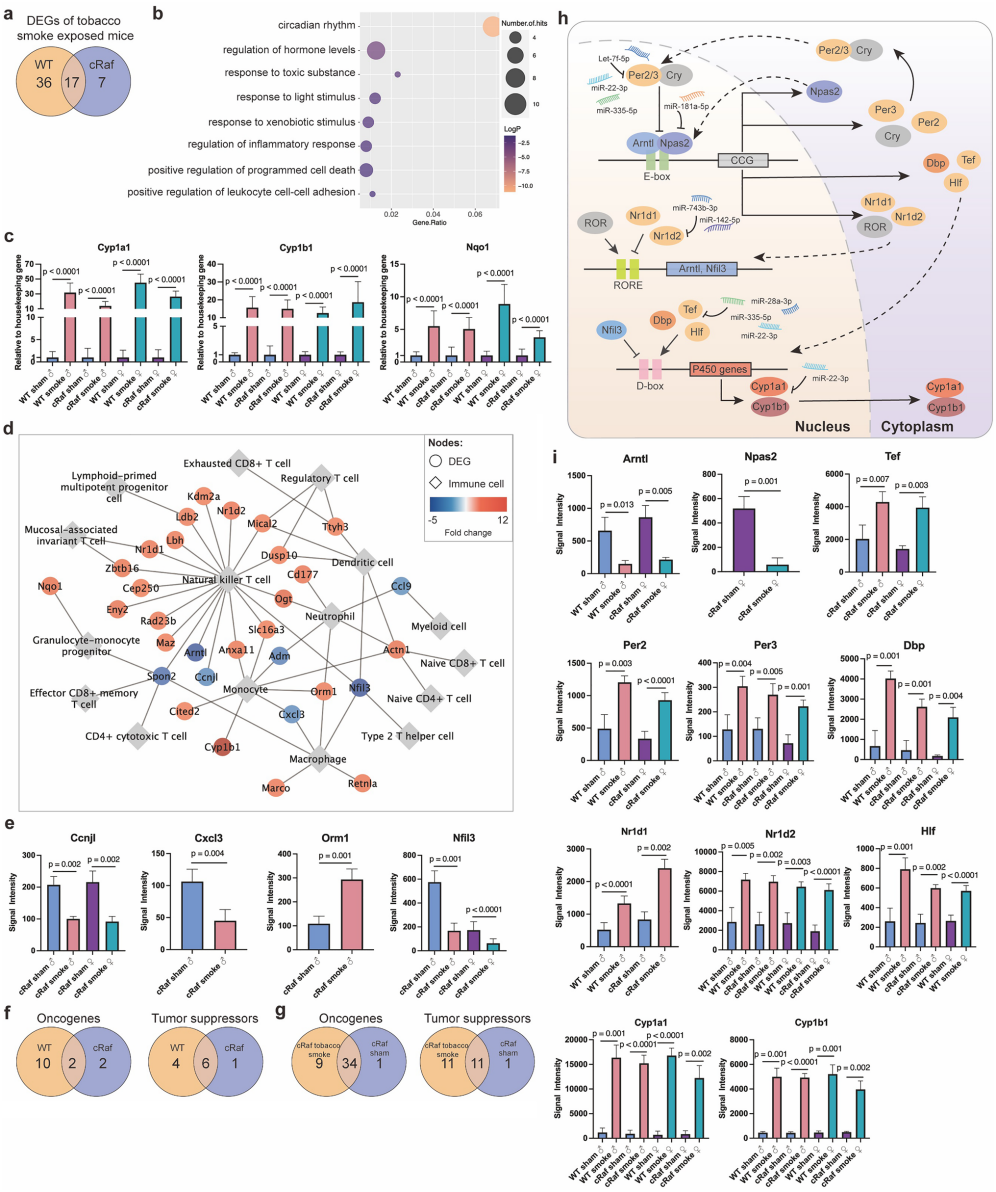
Genomic responses in lung tumors of tobacco smoke exposed animals. **a** Venn diagram of regulated genes in tobacco smoke exposed WT and cRaf transgenic mice. **b** Bubble-chart of enriched gene ontology terms for 60 tobacco smoke responsive genes. **c** RT-qPCR assays of Cyp1a1, Cyp1b1 and Nqo1. Two-tailed t test, error bar: 95% CI. **d** Regulation of immune cell marker genes in WT and transgenic mice exposed to tobacco smoke. **e** Examples of immune cell marker genes regulated in tobacco smoke exposed cRaf transgenic animals. Two-tailed t-test, error bar: 95% CI. **f** Venn diagram of regulated oncogenes and tumor suppressors: Comparison of tobacco smoke exposed WT and cRaf transgenic mice. **g** Venn diagrams of regulated oncogenes and tumor suppressors in sham and tobacco smoke exposed cRaf transgenic animals. **h** Schema of tobacco smoke induced regulation of circadian genes. **i** Histograms of regulated circadian genes. Two-tailed t-test, error bar: 95% CI. *WT* wild type, *DEG* differentially expressed gene

First, we compared WT tobacco smoke exposed mice to sham exposed controls. This defined 53 DEGs (49 up, four downregulated genes). Second, we compared cRaf tobacco smoke exposed mice to sham exposed cRaf transgenic controls, and this defined 24 DEGs (16 up, eight downregulated genes) of which 17 are in common between WT and cRaf tobacco smoke exposed animals (Fig. 2a). Despite varying genomic responses between WT and cRaf tobacco smoke exposed mice, the 17 commonly regulated genes mostly code for xenobiotic defense and circadian rhythm (Supplementary Table S3). Indeed, GO enrichment analysis of all 60 DEGs (Fig. 2a) emphasized circadian rhythm, regulation of hormone levels, inflammatory response and xenobiotic defense as significantly enriched terms (Fig. 2b). We observed marked inductions of the monooxygenases and xenobiotic defense enzymes *Cyp1a1*, *Cyp1b1* and NAD(P)H quinone oxidoreductase (*Nqo1*) following tobacco smoke exposure, and these were 24-, 11- and fourfold upregulated in tobacco smoke exposed female WT mice. Similar results were obtained for male WT mice and transgenic animals. To corroborate the findings by another method, we performed RT-qPCR assays and compiled the data of the two methods in Supplementary Table S2. As shown in Fig. 2c, we measured 31.7, 45.2, 15.6, 12.6, 5.5 and 8.9-fold changes, respectively, for *Cyp1a1*, *Cyp1b1 and Nqo1* in male and female WT animals. In the case of male and female cRaf transgenic mice we obtained 13.9, 26.4, 15.0, 18.7, 5.1 and 3.8-fold induced expression of these enzymes, and this emphasizes the robust effect of tobacco smoke on their regulation. Notwithstanding, qPCR assays tended to be more sensitive with higher fold changes in the regulation of xenobiotic defense genes when compared to the microarray platform. Notwithstanding, a comparison between WT and cRaf transgenic mice did not reach statistical significance.

#### Regulation of immune response genes

Strikingly, about half of the 60 DEGs (Fig. 2a) code for immune response (Supplementary Table S5), and Fig. 2d depicts their regulation in immune cells. We observed 18 and one immune response genes, respectively, that are uniquely upregulated in WT and cRaf transgenic animals (Supplementary Table S5 and Supplementary Fig. S3). Therefore, we observed major differences in the regulation of immune response genes following tobacco smoke exposure. Specifically, the majority of upregulated immune genes in WT mice code for negative regulation of cell migration and positive regulation of leukocyte activation. For instance, we observed fivefold induced expression of *O*-linked *N*-acetylglucosamine transferase, and this enzyme is upregulated in lung cancer (Mi et al. 2011) and was reported to promote an inflammatory phenotype of macrophages (Chang et al. 2020b). Conversely, with cRaf transgenic animals, orosomucoid 1 (*Orm1*) is the only immune response gene that is upregulated (threefold, Fig. 2e) and a recent study demonstrated Orm1 to enhance the immunosuppressive function of tumor-associated macrophages (TAMs) (Matsusaka et al. 2021). We observed repressed cyclin j like (Ccnjl), and the cyclin j-CDK complexes limit innate immune responses by reducing proinflammatory changes in macrophage metabolism, and its repression inhibits antitumor immunity (Chong et al. 2022). Similar, chemokine ligand 3 is repressed and this may be regarded as an adaptive response given its role in tumor growth (Chow and Luster 2014). Furthermore, the nearly 80% repression of nuclear factor, interleukin 3 regulated (*Nfil3*) in tobacco smoke exposed transgenic females is of great importance. This TF promotes the development of CD8α^+^ conventional dendritic cells (Kashiwada et al. 2011a), the NK cell lineage generation (Gascoyne et al. 2009), the innate lymphoid cell (Seillet et al. 2014) and stimulates type 2T-helper cell mediated cytokine production (Kashiwada et al. 2011b). *Nfil3* repression is part of an immune escape mechanism.

#### Regulation of oncogenes and tumor suppressors

To identify genes linked to the tumor promoting effects of tobacco smoke, we first considered DEGs derived from the comparison of sham and tobacco smoke exposed animals. We identified 12 and two oncogenes (13 up, one downregulated, Fig. 2f), respectively, in WT and cRAF tobacco smoke exposed animals in addition to 11 tumor suppressors (eight up, three downregulated, Supplementary Table S6). Although more oncogenes are upregulated in WT mice, the tumor promoting effects of tobacco smoke were only significant in cRaf transgenic animals (Fig. 1). Therefore, the tumor growth promoting effect of tobacco smoke was only visible in cRaf transgenic animals and differed by sex as described below. Similar results were obtained for the regulation of tumor suppressors, i.e., 10 (eight up, two downregulated) in WT and seven (four up, three downregulated) in cRaf transgenic animals with six genes regulated in common (Fig. 2f).

Given the difference in the regulation of oncogenes and tumor suppressors between WT and cRaf tobacco exposed animals, we investigated the combined effect of cRaf and tobacco smoke exposure. For this purpose, we considered DEGs derived from the comparison of cRaf tobacco smoke to WT sham exposed animals with DEGs derived from cRaf sham to WT sham exposed animals (Fig. 2g). This defined nine oncogenes (eight up, one downregulated) specifically regulated in tobacco smoke exposed transgenic animals while cRaf alone induced the regulation of 35 oncogenes. In the same comparison, 11 tumor suppressors were regulated of which six were down and 5 upregulated (Fig. 2g).

Specifically, we found the *Clock* paralogue gene neuronal PAS domain protein 2 (*Npas2*) almost silenced in tobacco smoke exposed cRaf animals, and the importance of this TF and tumor suppressor will be discussed below. Following tobacco smoke exposure of WT mice, we observed two to threefold upregulation of cathepsin k, tetraspanin 4 (*Tspan4*) and *Rgl1*. The coded proteins promote LC, cell proliferation and invasion by stimulating mTOR and Ras signaling pathways (Yang et al. 2020; Deng et al. 2021; Feig 2003; Li et al. 2019a). Likewise, tobacco smoke exposure of WT male mice resulted in two to threefold induced expression of tumor suppressors, notably *Acer2*, *Lbh* and *Zbtb16*, and are reported to inhibit tumor growth and to induce apoptosis in LC (Wang et al. 2013, 2017a; Deng et al. 2018). Therefore, adaptive responses to tobacco smoke included the upregulation of tumor suppressors in males but not females.

As shown in Fig. 2a we identified 53 and 24 genes, respectively, in tobacco smoke exposed WT and cRaf transgenic animals of which nearly one-half code for the circadian clock. Furthermore, there are 3 clock genes uniquely regulated in cRaf tobacco smoke exposed mice, i.e., *Nfil3*, *Npas2* and thyrotroph embryonic factor (*Tef*). Together this emphasizes the strong effect of tobacco smoke on the circadian clock as discussed below.

#### Tobacco smoke perturbs the circadian clock

We identified several basic helix–loop–helix/Per-ARNT-SIM (bHLH-PAS) TFs and regulators of the circadian clock as significantly upregulated in response to tobacco smoke exposure. This included nuclear receptor subfamily 1 group D member 1 and 2 (*Nr1d1, Nr1d2*), (also known as Rev-Erbα/Erbß) and period circadian regulator 2 and 3 (*Per2, Per3*). Conversely, *Npas2* and the aryl hydrocarbon receptor nuclear translocator-like (*Arntl* alias *Bmal1*) are repressed. Essentially, all these TF function in a regulatory loop (Fig. 2h) whereby Per2/3 and Cry inhibit Arntl/Npas2-dependent promoter activation of circadian clock genes. Moreover, Nr1d1 and Nr1d2 are transcriptional repressors of *Arntl*. Thus, their induced expression is a likely cause of repressed *Arntl* gene transcription in tobacco smoke exposed animals. Furthermore, upregulation of *Per2/3* blocked Arntl/Npas2-dependent activation of circadian clock genes even though some of the circadian clock target genes were upregulated, including *Tef* and the D site albumin promoter binding protein (*Dbp*). These TFs bind to the D-box element in the promoter of the *Cyp1b1* gene to stimulate its expression. Additionally, *Nfil3*, i.e., a transcriptional repressor of the *Cyp1b1* and we observed its up to fivefold repression in cRaf transgenic mice whereas its target *Cyp1b1* was up to 11-fold upregulated. This demonstrates the importance of the bHLH–PAS heterodimers in augmenting expression of xenobiotic defense genes following tobaccos smoke exposure. Collectively, we obtained evidence for tobacco smoke to disturb the circadian clock, and this included the repression of the tumor suppressor Arntl and the upregulation of the *Nr1d2* oncogene.

Repression of *Arntl/Bmal1* is of fundamental importance in the control of the circadian rhythm, and we identified two ways by which tobacco smoke exposure caused repression of *Arntl/Bmal1*, i.e., by blocking its TF activity via induced expression of *Per2/3* repressor activity, and transcriptionally through binding of the Nr1d1 and Nr1d2 repressors to the RORE binding site in the promoter of the *Arntl/Bmal1* gene (Fig. 2h). We highlight significantly regulated circadian genes in Fig. 2i.

#### Regulation of oncomirs and tumor suppressors

**Fig. 3:**
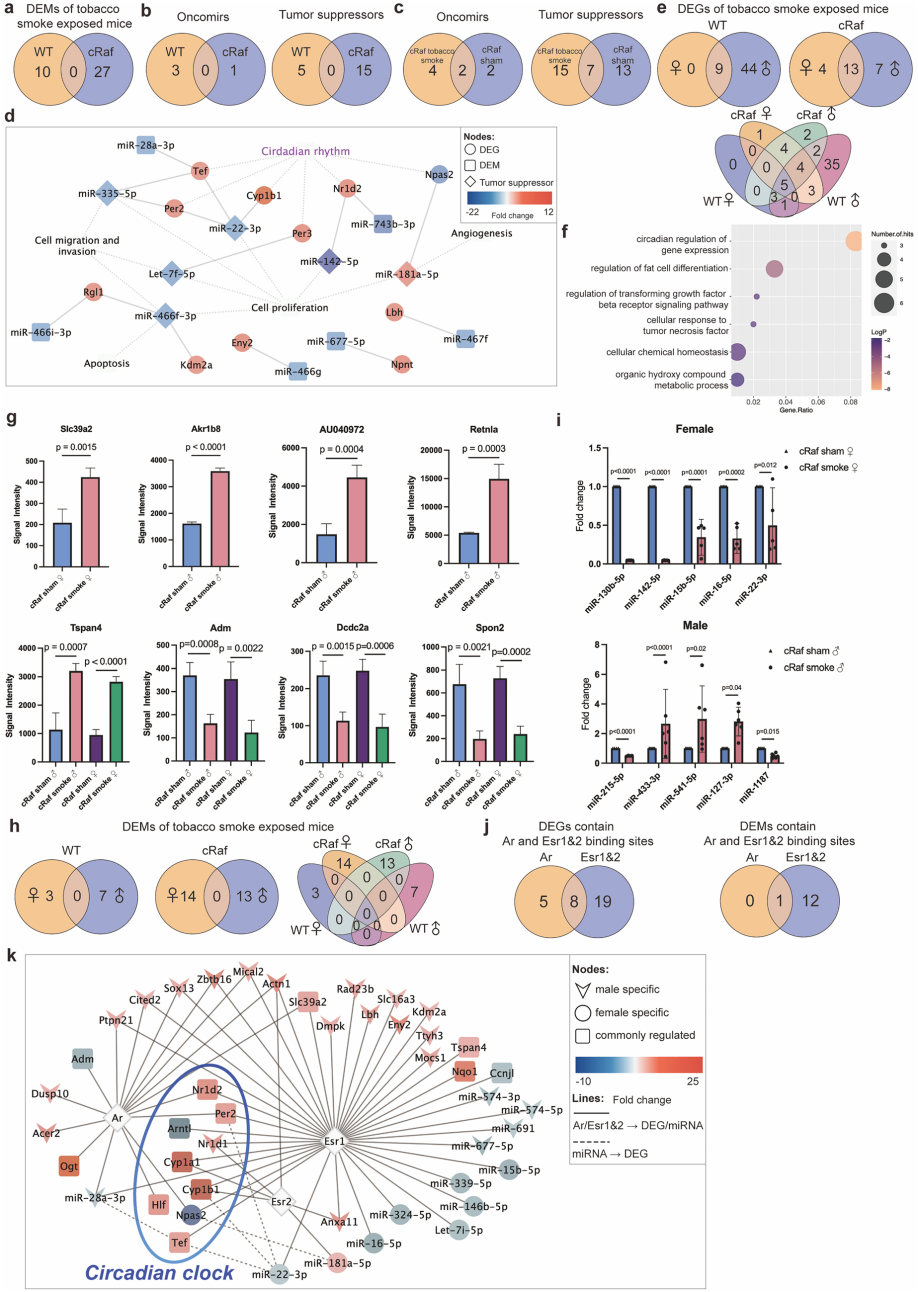
Tobacco smoke induced expression of miRNAs and sex-dependent genomic responses. **a** Venn diagram of DEMs in tobacco smoke exposed WT and cRaf transgenic mice. **b** Venn diagrams of oncomirs and tumor suppressors in tobacco smoke exposed WT and cRaf transgenic mice. **c** Venn diagrams of oncomirs and tumor suppressors in cRaf sham and tobacco smoke exposed transgenic animals. **d** MiRNA-gene regulatory network in tobacco smoke exposed mice. The network consisted of 11 DEGs and 12 miRNAs, and we highlight the functions of miRNAs. **e** Venn diagrams of sex-specific regulations of DEGs in WT and cRaf transgenic mice following tobacco smoke exposure. **f** Bubble-chart of enriched ontology terms for 44 DEGs specifically regulated in WT males. **g** Histograms of sex-specific and common regulations of DEGs in cRaf transgenic animals. Two-tailed t-test, error bar: 95% CI. **h** Venn diagrams of sex-specific DEMs in WT and cRaf transgenic mice. **i** Scatter plots of sex-specific regulations of DEMs in cRaf transgenic animals. Two-tailed t-test, error bar: 95% CI. **j** Venn diagrams of DEGs and DEMs containing androgen and estrogen receptor binding sites. **k** Network of androgen and estrogen receptor regulated genes following tobacco smoke exposure. The network consists of 13 DEMs and 32 DEGs, which contain transcription factor binding sites for androgen and estrogen receptors. *WT* wild type, *DEG* differentially expressed gene, *DEM* differentially expressed miRNA, *Ar* androgen receptor, *Esr1* estrogen receptor 1, *Esr2* estrogen receptor 2

We compared the genomic data of tobacco smoke exposed WT and cRaf transgenic mice to sham exposed animals. This defined 37 DEMs of which 10 (downregulated) and 27 (eight up, 19 downregulated), were regulated in WT and cRaf mice. As shown in Fig. 3a none of the DEMs are common between WT and transgenic animals (Supplementary Table S3) and, therefore, the regulatory gene-networks differ between WT and cRaf animals.

Regarding the functions of regulated miRNAs, > 60% code for tumor suppressors and oncomirs (Fig. 3b). We considered the downregulation of oncomirs as an adaptive response to tobacco smoke. For instance, we found miR-151-5p > twofold repressed in WT females. This oncomir promotes cell proliferation, migration and invasion in LC (Daugaard et al. 2017). Similar, miR-130b-5p, miR-15b-5p and miR-574-5p were significantly repressed in cRaf mice (range two to 21-fold), and these miRNAs promote cell proliferation and metastasis (Kim et al. 2021a; Wang et al. 2017b; Zhou et al. 2016). Of the 37 DEMs (Fig. 3a) twenty function as tumor suppressors (Supplementary Table S6) of which 15 (five up, 10 downregulated) were regulated in cRaf transgenic animals. The repression of so many tumor suppressors documents the complex responses of the pulmonary genome to the detrimental effects of tobacco smoke exposure. Specifically, let-7d-5p, let-7f-5p and let-7i-5p are members of the tumor suppressor let-7 family and their two to fourfold repression impaired  tumor suppressive functions in cRaf tobacco smoke exposed females. Indeed, let-7 inhibits LC tumor growth by targeting c-MYC, KRAS and HMGA2 (Johnson et al. 2005; Kolenda et al. 2014), binds to DICER, i.e., a RNAase which processes miRNAs, and induces cell autophagy in LC (Li et al. 2021). Strikingly, the aforementioned tumor suppressors remained highly expressed in tobacco smoke exposed males, and the sex-specific repression of let-7 is a likely reason for an increased tumor burden observed in females. Tobacco smoke exposure of cRaf transgenic mice also caused significant repression of miR-142-5p, miR-146b-5p, miR-16-5p, miR-200b-5p, miR-215-5p, miR-22-3p and miR-335-5p (range 2 to 21-fold), and these tumor suppressors inhibit tumorigenesis, cell proliferation, migration, and enhance sensitivity to cisplatin treatment (Supplementary Table S6). For instance, tobacco smoke exposure of cRaf females caused repression of the tumor suppressor miR-335-5p, and nearly silenced the tumor suppressor miR-142-5p. Moreover, in tobacco smoke exposed WT mice, miR-1224-5p, miR-30c-2-3p, miR-339-5p and miR-466f-3p are two to fourfold repressed, and these tumor suppressors inhibit LC cell proliferation, migration and invasion, and induce apoptosis (Wang et al. 2018; Tong et al. 2018; Li et al. 2020; Shi et al. 2020; Yao et al. 2018; Zhong et al. 2014). Therefore, tobacco smoke exposure caused repression of tumor suppressors in non-transgenic animals as well. Notwithstanding, miR-541-5p, miR-127-3p, miR-433-3p, miR-211-5p and miR-181a-5p were two to threefold upregulated and these tumor suppressors inhibit LC tumor growth, invasion, and angiogenesis (Xu et al. 2018; Li et al. 2019b; Bi et al. 2016; Ma et al. 2015; Quan et al. 2018).

Collectively, tobacco smoke exposure resulted in complex regulations of tumor suppressors and oncomirs, and the responses differed between WT and transgenic mice. The Venn diagram shown in Fig. 3c highlights the additional regulation of oncomirs and tumor suppressors following tobacco smoke exposure. Here, we compared DEMs derived from the comparison of cRaf tobacco smoke exposed mice to WT sham animals with DEMs derived from the comparison of cRaf sham versus WT sham.

#### MiRNA-gene networks

To determine miRNA-gene interactions, we evaluated published experimental evidence and only considered validated, i.e., cross-linked and immunoprecipitated targets. This defined 11 DEGs targeted by 12 miRNAs (Fig. 3d). We identified seven miRNAs of which six were repressed (Supplementary Table S3). Remarkably, tobacco smoke exposure of transgenic females caused repression of several tumor suppressors and included let-7f-5p while its circadian clock target gene *Per3* was upregulated (Chirshev et al. 2019). Likewise, the tumor suppressor miR-335-5p was significantly repressed while its target genes *Per2* and *Tef* were upregulated and this tumor suppressor regulates cell cycle and epithelial-mesenchymal transition (Du et al. 2019; Wang et al. 2020). The tumor suppressor miR-142-5p was nearly silenced to < 5% of controls, and this miRNA targets PI3K catalytic subunit alpha which is commonly deregulated in cancers (Wang et al. 2017c). As shown in Fig. 3d, *Nr1d2* is a target of miR-142-5p and miR-743b-3p, and another genomic study showed multiple circadian genes including *Nr1d2* to be specifically associated with increased risk of human LC (Mocellin et al. 2018). Furthermore, induced expression of miR-181a-5p caused repression of the tumor suppressor *Npas2* in tobacco smoke exposed cRaf females (Ma et al. 2015).

### Sex-dependent genomic responses in WT and cRaf tobacco smoke exposed animals

We investigated sex-specific responses in WT and cRaf tobacco smoke exposed mice, and the results are given in Fig. 3e. With WT mice, of the 53 responsive genes, 44 (40 up, four downregulated) are male specific. Conversely, none were female specific and 9 were regulated in common. The GO enrichment analysis of tobacco smoke exposed WT males defined circadian regulation of gene expression, regulation of fat cell differentiation, regulation of transforming growth factor beta receptor signaling and cellular chemical homeostasis as significantly enriched terms (Fig. 3f). Tobacco smoke exposure of WT females did not elicit sex-specific gene regulations which is astonishing. Conversely, we identified 4 genes uniquely regulated in cRaf tobacco smoke exposed females (Fig. 3e), of which *Per2* and the solute carrier family 39 were upregulated (range two to threefold) whereas *Arntl* and *Npas2* were repressed (four and ninefold). The upregulation of solute carrier family 39 is an interesting finding, and likely caused by cadmium, which is a well characterized constituent of tobacco smoke. We summarize in Fig. 3g the sex-specific regulation of genes in tobacco smoke exposed transgenic animals. Notwithstanding and irrespective of sex and transgenicity, there are five genes regulated in common, and these code for xenobiotic defense and components of the circadian clock, i.e., C*yp1a1, Cyp1b1, Nqo1, Tspan4* and *Nr1d2* (Fig. 3e).

We also searched for sex-dependent regulation of miRNAs. Strikingly, none were common when WT and cRaf transgenic animals were compared (Fig. 3h), and this underscores the strict sex-dependent regulation of miRNA in tobacco smoke exposed animals. Specifically, we identified 3 repressed DEMs in tobacco smoke exposed WT females of which miR-30c-2-3p and miR-339-5p function as tumor suppressors by targeting Rho GTPase activating protein 11A (Zheng et al. 2022), and BCL6 (Li et al. 2018), and repression of miR-151-5p inhibits cell proliferation in LC (Daugaard et al. 2017). In the same comparison we identified 7 DEMs specifically repressed in WT males and this included two to fourfold repressed miR-1224-5p, miR-574-3p and miR-466f-3p. These tumor suppressors inhibit cell proliferation by targeting matrix metallopeptidase 3 (Zheng et al. 2019), inhibit metastasis (Tong et al. 2018; Li et al. 2020; Shi et al. 2020; Yao et al. 2018), and induce apoptosis (Tong et al. 2018).

Tobacco smoke exposure of cRaf females caused the regulation of 14 miRNAs (1 up, 13 downregulated) of which 10 and 2, respectively, code for tumor suppressors and oncomirs (Supplementary Table S7). Of the tumor suppressors, 9 were downregulated (range 2- to 21-fold) and, therefore, support tumor growth. However, the oncomirs miR-130b-5p and miR-15b-5p were repressed and this highlights the complex interplay of cRaf and tobacco smoke exposures in female transgenic mice. Examples of highly repressed tumor suppressors include miR-16-5p which stimulates cell proliferation by regulating MEK1 activity (Chen et al. 2019). Another example relates to the twofold repression of miR-22-3p and this tumor suppressor regulates cell growth through MET/STAT3 signaling (Yang et al. 2021). Importantly, the estrogen receptor inhibits miR-22-3p promoter activity and its consequences will be discussed in the next paragraph.

Meanwhile, tobacco smoke exposure of transgenic males resulted in 13 regulated miRNAs (7 up, 6 down) (Fig. 3h), of which 5 (4 up, 1 downregulated) are tumor suppressors in addition to one oncomir. These miRNAs take part in the control of cell proliferation, migration and invasion (Supplementary Table S6), and given the upregulation of tumor suppressors, the data infer male animals to be partially protected against the detrimental effects of cRaf transgenicity and tobacco smoke exposure.

Depicted in Fig. 3i are examples of sex-specific regulations of miRNAs in transgenic animals exposed to tobacco smoke. Together, we identified 9 repressed tumor suppressors in cRaf females exposed to tobacco smoke, and this provides a molecular rationale for increased tumor growth seen among these animals (Fig. 1). Conversely, with male transgenic mice only one tumor suppressor was repressed following tobacco smoke exposure, i.e., miR-215-5p, and this miRNA inhibits proliferation and migration of LC cells (Cai et al. 2017).

#### The role of the estrogen and androgen receptor in sex-specific genomic responses to tobacco smoke exposures

To understand sex-specific regulations of DEMs and DEGs, we searched for targets regulated by the estrogen (*Esr1* and *Esr2*) and androgen receptor (*Ar*). We queried the GSEA, Transmir v2.0 and hTFtarget databases and considered chromatin IP proven binding sites in promoters of DEGs and DEMs. As detailed above, tobacco smoke exposure caused 60 DEGs (Supplementary Table S3) of which 53% are targets of sex hormone receptors (Supplementary Table S7). We identified 19 and 5 DEGs, respectively, as targets of the estrogen and androgen receptors, while an additional 8 DEGs contained binding sites for both hormone receptors (Supplementary Table S7, Fig. 3j). However, the genes coding for the *Esr1&2* and *Ar* receptors were not regulated.

Tobacco smoke exposure of WT females caused upregulation of 9 genes but none were sex specific, i.e., these were also regulated in WT males. Additionally, we identified estrogen receptor binding sites in the promoters of the four DEGs specifically regulated in cRaf females, i.e., the clock genes *Npas2, Per2, Arntl* and the cadmium transporter *Slc39a2*. Therefore, a direct relationship between hormone receptor stimulated promoter activity and the regulation of these genes can be inferred. Furthermore, we identified 5 genes regulated in common (*Cyp1a1, Cyp1b1, Nqo1, Nr1d2 and Tspan4*) (Fig. 3e), and although all of them contain estrogen receptor binding sites (Fig. 3k), their promoters also contain binding sites for the aryl-hydrocarbon receptor (Ahr) (Supplementary Table S8). Given their likewise regulation in tobacco smoke exposed males and females, we assume a prominent role of the Ahr and a lesser role of the steroid hormone receptors in the regulation of these xenobiotic defense genes.

Subsequently, we searched for hormone receptor binding sites in promoters of DEMs, and this revealed 13 miRNA targets of the estrogen receptors of which miR-28a-3p is a target of both the estrogen and androgen hormone receptors (Fig. 3k, Supplementary Table S7). Specifically, of the estrogen-responsive miRNAs, tumor suppressors let-7i-5p, miR-146b-5p, miR-16-5p, miR-22-3p and miR-339-5p were uniquely repressed (two to threefold) in females, and Fig. 3k shows the complex interplay between miRNAs and genes targeted by sex hormone receptors.

The highly regulated target genes *Cyp1a1* and *Cyp1b1* are targets of the estrogen and aryl hydrocarbon receptors. Typically, these cytochrome monooxygenases are highly induced in response to tobacco smoke exposure and are of key importance in the metabolic conversion of tobacco smoke constituents into carcinogenic compounds. In a previous study with human volunteers, we demonstrated significantly induced *Cyp1a1* and *Cyp1b1* transcripts in bronchial biopsies taken distal from the carina of the left upper lobe (Thum et al. 2006). Strikingly, independent research demonstrated a 6.2-fold induced CYP1A1 protein expression (*p* < 0.01) following treatment of normal human bronchial epithelial cells with 17β-estradiol (estrogen receptor agonist) whereas a defined cigarette smoke extract induced CYP1A1 by just twofold (Han et al. 2005). Additionally, a clinical study revealed significantly higher CYP1A1 expression among female smoker LC patients as compared to male LC smokers (Mollerup et al. 1999). Altogether, the data suggest the estrogen receptor to be a critical factor in *CYP1A1* expression (Honkakoski and Negishi 2000). Similarly, *Cyp1b1* is a transcriptional target of the estrogen receptor and this monooxygenase participates in the transformation of estradiol to its genotoxic metabolites 4-hydroxyestrogen and 4-hydroxyestradiol. Therefore, next to its role in the production of carcinogenic metabolites derived from tobacco smoke constituents, the marked upregulation of *Cyp1b1* likely increased the production of estradiol genotoxic metabolites to promote tumorigenesis (Słowikowski et al. 2017). Additionally, mice exposed to tobacco smoke caused an average threefold induction of *Tef* and Hepatic leukemia factor (*Hlf*), and these code for TFs which stimulate Cyp1a1 and Cyp1b1 promoter activity as well (Fig. 2h).

In regards to the estrogen receptor and its role in the regulation of miRNAs, we wish to emphasize miR-22-3p. This miRNA is a direct target of estrogen receptor, and we noticed its downregulation following tobacco smoke exposure. Correspondingly, its gene targets were upregulated (Fig. 3k). The target genes code for clock genes, and this underscores the effects of tobacco smoke on the circadian rhythm. MiR-22-3p functions as a tumor suppressor and is repressed in LC. A recent study revealed transfection of miR-22-3p agomirs to inhibit cell proliferation of human LC cell lines (Yang et al. 2021). Overall, we propose a regulatory loop whereby tobacco smoke represses miR-22-3p, which in turn dysregulates circadian genes and their regulation by the steroid hormone receptor. Moreover, exposure to tobacco smoke enhanced transcription of *Cyp1a1* and *Cyp1b1*, which reinforces the production of carcinogenic metabolites especially among females.

### The combined effect of cRaf and tobacco smoke on the pulmonary genome

To identify genes regulated by the two effectors, i.e., cRaf and tobacco smoke, we compared the genomes of cRaf tobacco smoke males and females with their corresponding WT sham exposed controls. This defined 158 DEGs of which 99 were up and 59 downregulated (Supplementary Table S3).

**Fig. 4:**
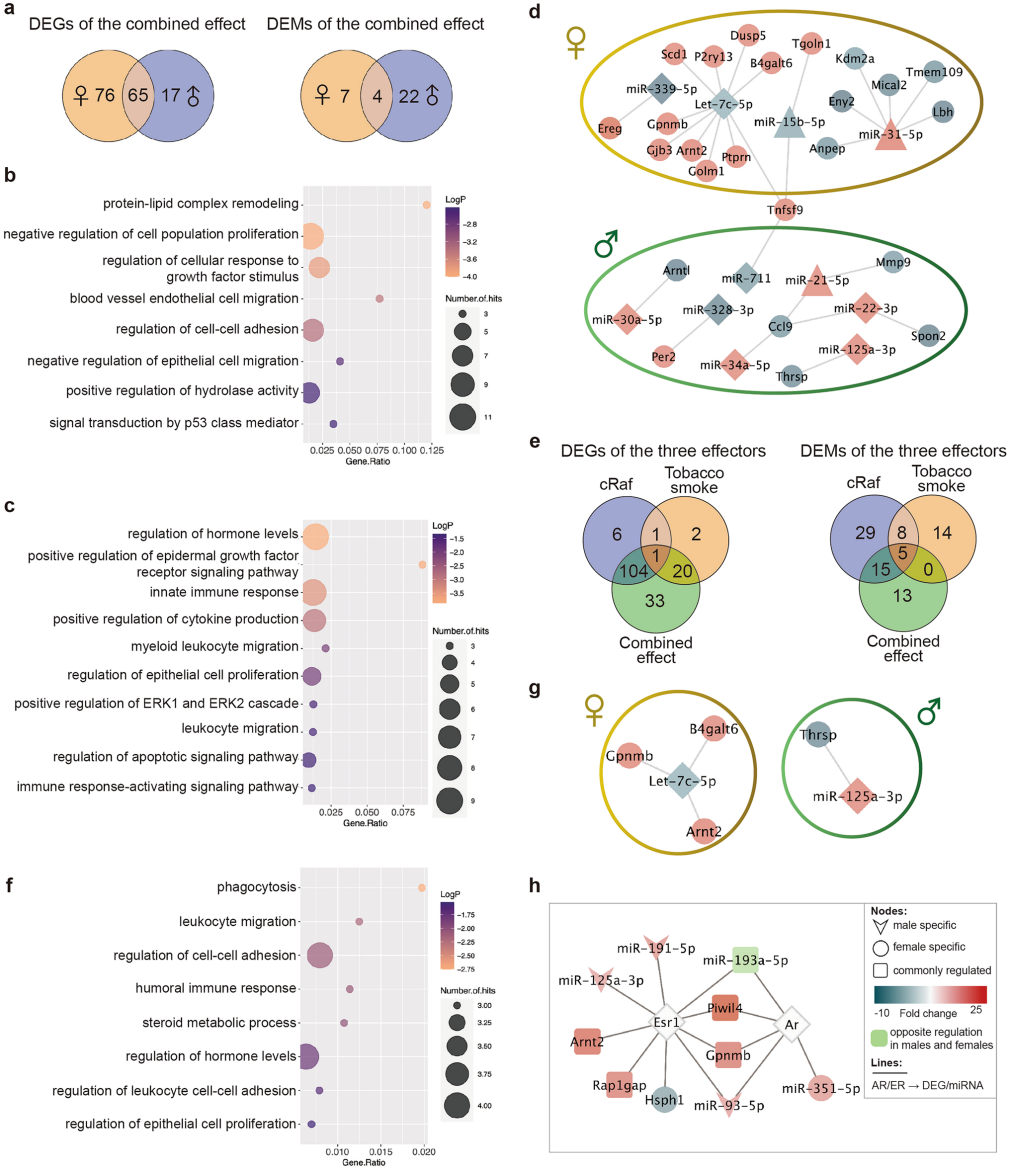
cRaf—tobacco smoke interactions. **a** Venn diagrams of sex-specific DEGs and DEMs in transgenic mice exposed to tobacco smoke. **b** Bubble chart of significantly enriched ontology terms for 76 female-specific DEGs. **c** Bubble chart of significantly enriched ontology terms for 65 common DEGs. **d** MiRNA-gene regulatory network of sex-specific gene regulations in tobacco smoke exposed transgenic mice. **e** Venn diagrams of DEGs and DEMs of the three effectors, i.e., cRaf, tobacco smoke, and the combined effect. **f** Bubble chart of significantly enriched ontology terms for 33 DEGs regulated by the combined effect. **g** MiRNA-gene networks of genes regulated by the combined effect. Shown are the miRNAs and genes regulated by combined effect. Note the sex-specific regulations. **h** MiRNA-gene networks of genes targeted by the androgen and estrogen receptor. Shown are miRNAs and genes uniquely regulated by the combined effect. *DEG* differentially expressed gene, *DEM* differentially expressed miRNA

Of the 158 DEGs, 76 and 17 are female and male specific while 65 DEGs are regulated in common (Fig. 4a, Supplementary Table S3). In regards to the female-specific DEGs, the GO enrichment analysis defined regulation of cellular response to growth factor stimulus, regulation of cell–cell adhesion, signal transduction by p53 and protein–lipid complex remodeling as significantly enriched terms (Fig. 4b). For the common DEGs significantly enriched terms were positive regulation of cytokine production, myeloid leukocyte migration, regulation of epithelial cell proliferation, regulation of ERK signaling and apoptotic signaling pathways (Fig. 4c). However, the number of male specific DEGs was too small to define meaningful GO terms. Among the commonly regulated genes, we wish to highlight gastrokine 2, granzyme E and Meg3, which were 28-, 81-, 4-fold upregulated in transgenic males. Conversely, repetin, which codes for epidermal differentiation was fivefold repressed. Note, these genes were also regulated in transgenic females, however to a lesser extent, i.e., eight, ten and threefold upregulated and twofold repressed, respectively. Gastrokine 2 stimulates apoptosis of gastric cancer cells by inhibiting NF-κB signaling and by activating JNK signaling pathway (Menheniott et al. 2016; Zhang et al. 2019a), while granzymes are expressed on cytotoxic T lymphocytes and NK cells to induce cell death (Voskoboinik et al. 2015). Meg3 is a tumor suppressor which was reported to be downregulated in LC (Ghafouri-Fard and Taheri 2019). Therefore, we regard its fourfold upregulation in tobacco smoke exposed transgenic animals (both sexes) as an adaptive response to the detrimental effects of cRaf hyperactivity and tobacco smoke. Moreover, Meg3 is also upregulated in sham exposed transgenic animals (Zhong and Borlak 2023), while the repression of repetin in transgenic males and females supports epithelial cell dedifferentiation (Supplementary Table S3) (Huber et al. 2005; Krieg et al. 1997). Interestingly, in sham exposed transgenic males but not in females, gastrokine 2 is highly induced (17-fold). Furthermore, granzyme E is not regulated in sham exposed animals, and this emphasizes an activation of a caspase-independent death program in tobacco smoke exposed animals.

The same comparison defined 33 DEMs, of which 7 and 22, respectively, were female and male specific. Furthermore, 4 miRNAs were regulated in common (Fig. 4a) and included an up to fivefold and 28-fold induced expression of the oncomir miR-31-5p and tumor suppressor miR-127-3p (Supplementary Table S3 and S6). Additionally, tumor suppressor miR-193a-5p and miR-30c-2-3p were up to threefold induced in males; but two and fourfold repressed in females (Supplementary Table S3). Importantly, miR-193-5p suppressed metastasis by targeting PIK3R3 and mTOR in LC (Yu et al. 2015), and repressed expression of miR-30c-2-3p induced EMT in LC (Zhong et al. 2014). Together, we demonstrate opposite regulation of tumor suppressors in females with males being partly protected against the detrimental effects of tobacco smoke and cRaf. Therefore, our findings provide a molecular rationale for the significantly increased tumor growth in cRaf females exposed to tobacco smoke (Fig. 1).

To construct miRNA-gene networks, we considered cross-linked miRNA immunoprecipitated targets. This allowed us to build a network consisting of 24 DEGs which are targeted by 11 miRNAs. As shown in Fig. 4d, we obtained sex-specific networks. For instance, tumor suppressor let-7c-5p and miR-339-5p were specifically repressed in females and these regulate EMT (Li et al. 2014), induce apoptosis (Liu et al. 2022), and increase radio-sensitivity in LC (Wang et al. 2018). Their target genes were upregulated. Conversely, miR-30a-5p, miR-125a-3p, miR-34a-5p and miR-22-3p were specifically upregulated in males, and their target genes were downregulated. These tumor suppressors inhibit cell proliferation (Yang et al. 2021; Li et al. 2015a), migration and invasion (Li et al. 2015a; Zhang et al. 2019b), and induce apoptosis in LC (Zhang et al. 2019b; Aida et al. 2021; Quan et al. 2019).

#### DEGs and DEMs uniquely regulated in tobacco smoke exposed transgenic animals

As depicted in Fig. 4e, there are 33 DEGs and 13 miRNAs uniquely regulated by the combined effect of cRaf and tobacco smoke; however, were not regulated by either effector. The DEGs result from the combined effect function in phagocytosis, leukocyte migration, cell–cell adhesion, epithelial cell proliferation and steroid metabolic process (Fig. 4f). For instance, Schlafen 4, an interferon responsive gene, which is mouse specific but has a human orthologue (Schlafen 12L) was threefold repressed in tobacco smoke exposed cRaf transgenic males. Although the roles of Schlafen proteins in cancer biology are not clearly defined there is sufficient evidence for these nucleic acid Zink-finger binding proteins to inhibit cell growth (Schwarz et al. 1998). Thus, its repression will support cell growth and the current knowledge of Schlafen 4 is suggestive to influence T-cell and macrophage function (Al-Marsoummi et al. 2021). Further examples include twofold upregulated lipocalin 2 in males, and this acute-phase protein is produced by granulocytes, macrophages and epithelial cells, and is part of the innate immune system to support pro- and anti-inflammatory responses (Guardado et al. 2021). Conversely, the immunoglobulin heavy chain was twofold repressed, and mice deficient of immunoglobulin heavy chain joining region do not produce B cells (Kitamura et al. 1991). Moreover, the HtrA serine peptidase 3 (Htra3), i.e., a pro-apoptotic protease was threefold repressed in females. This tumor suppressor has been reported to be downregulated in various cancers including LC (Wenta et al. 2019). A further highly interesting result relate to an induced expression of WAP four-disulfide core domain 17 in cRaf tobacco smoke exposed females. This protein stimulates the immune-suppressive activity of polymorphonuclear myeloid-derived suppressor cells and the antigen cross-presentation by dendritic cells in cancers (Veglia et al. 2021). It is part of an immune escape mechanism.

Of the 13 uniquely regulated miRNAs, 10 function as tumor suppressors, of which 2 were specifically repressed in females (let-7c-5p, miR-150-5p), while 6 were specifically regulated in males, i.e., miR-125-3p, miR-191-5p, miR-200c-3p, miR-30a-5p and miR-361-5p were up-, and miR-181a-1-3p was downregulated. Furthermore, miR-193a-5p was upregulated in males but downregulated in females. These tumor suppressors inhibit cell proliferation, metastasis and induce apoptosis in LC (Supplementary Table S6). We also identified miR-93-5p as twofold upregulated in males, and this oncomir promotes cell proliferation and migration in LC (Yang et al. 2018). Depicted in Fig. 4g is the miRNA-gene network of the DEGs and miRNAs specifically regulated by the combined effect.

Among the genes and miRNAs uniquely regulated by the combined effect, we identified 5 DEGs and 5 DEMs as targets of the sex hormone receptors (Fig. 4h). The three to fourfold upregulation of aryl hydrocarbon receptor nuclear translocator 2 (*Arnt2*) is an Esr1 target gene. This nuclear translocator forms a heterodimer with the activated Ahr, and regulates gene expression such as *Cyp1a1* and *Cyp1b1* (Choudhary and Malek 2020), which we found highly induced as detailed above (Supplementary Table S3).

### Clinical translation and sex-specific genomic responses to tobacco product use

**Fig. 5:**
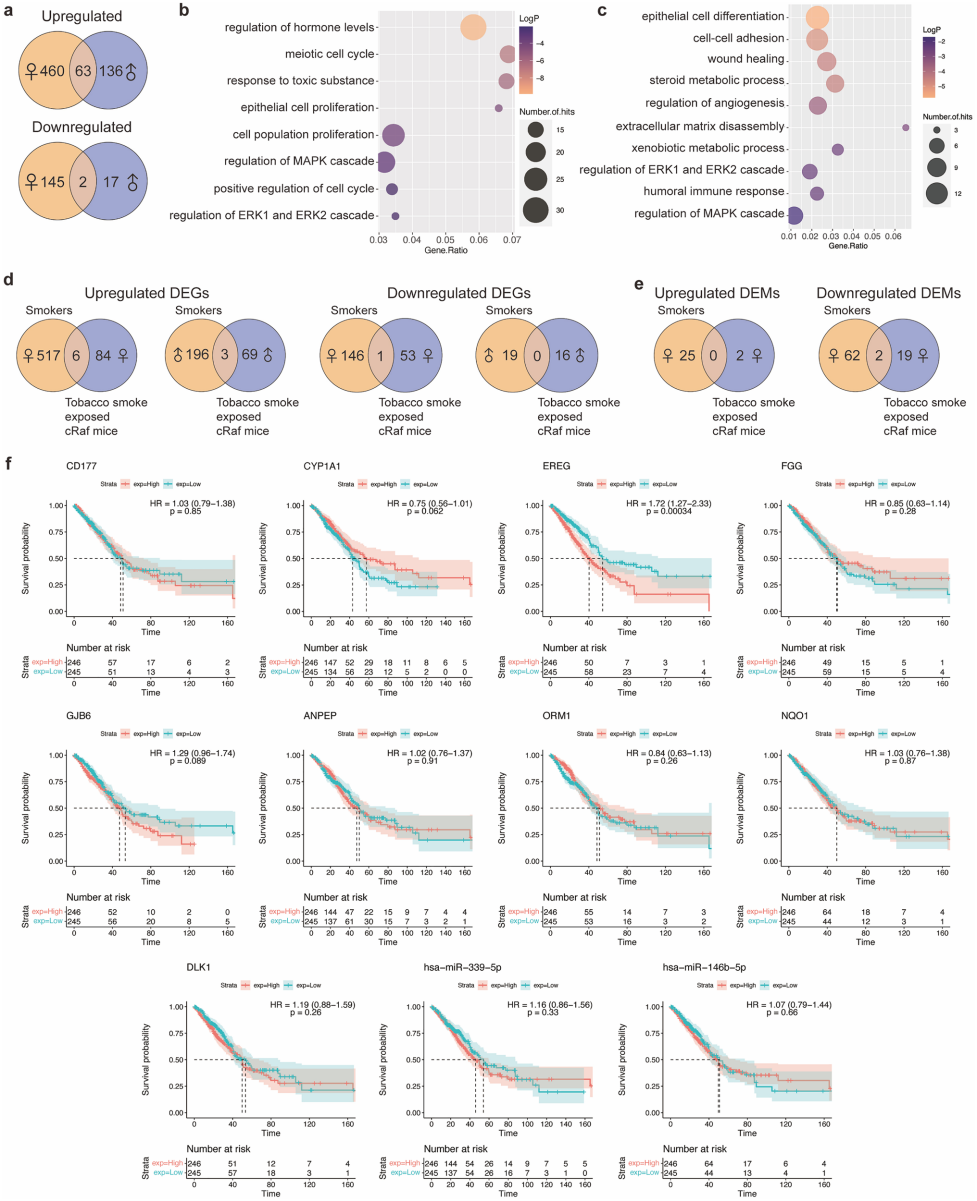
Genomic responses of smokers in human lung adenocarcinoma. **a** Venn diagrams of sex-specific DEGs in LC patients. **b**, **c** Significantly enriched ontology terms of female-specific upregulated (**b**) and downregulated (**c**) DEGs. **d** Clinical validation of sex-specific DEGs identified in lung tumors of tobacco smoke exposed cRaf transgenic mice. The Venn diagrams show the commonly regulated DEGs between smokers and cRaf transgenic mice. **e** Clinical validation of sex-specific DEMs identified in lung tumors of tobacco smoke exposed cRaf transgenic mice. The Venn diagrams show the commonly regulated DEMs between female smokers and female tobacco smoke exposed cRaf transgenic mice. **f** Kaplan–Meier survival plots for DEGs and DEMs commonly regulated in cigarette smoking LC patients and tobacco smoke exposed cRaf transgenic mice. *DEG* differentially expressed gene, *DEM* differentially expressed miRNA

Based on the set paradigm of our study, we compared LC patients in regards to sex and tobacco product use history (Supplementary Table S9). This defined 670 (523 up, 147 downregulated) and 218 (199 up-, 19 down-regulated) DEGs in females and males. Subsequently, we compared DEGs of female and male patients. This defined 605 (460 up and 145 downregulated) genes uniquely regulated in female smokers, while 153 DEGs (136 up and 17 downregulated) were regulated in male smokers only (Fig. 5a, supplementary Table S9). The genomic data infer females to be much more responsive to the effects of tobacco smoke exposure with the regulation of an additional 452 genes, and similar results were obtained with transgenic females, i.e., the total number of DEGs increased by fourfold.

Of the uniquely upregulated genes in female smokers, enriched GO terms are epithelial cell differentiation, regulation of hormone levels, meiotic cell cycle, and response to toxic substance (Fig. 5b). Vice versa, for repressed DEGs, significantly enriched terms are epithelial cell differentiation, wound healing, regulation of angiogenesis and extracellular matrix disassembly (Fig. 5c). Note, for male LC patients the Metascape analysis did not lead to meaningful terms.

Shown in Fig. 5d are common and uniquely regulated genes linked to tobacco smoke exposure and sex. In this comparison, we addressed the question whether genes regulated in TS exposed transgenic animals were also regulated in LC patients (smokers). We identified seven DEGs (six up and one down) as commonly regulated between cRaf females and female LC patients exposed to tobacco smoke (Supplementary Table S9). Specifically, CYP1A1 is a monooxygenase that catalyzes the production of reactive metabolites of tobacco smoke components and was highly induced, i.e., > 24-fold and > eightfold, respectively, in mice and female LC patients (Supplementary Table S9). Likewise, *NQO1* catalyzes the reduction of reactive quinones and through redox cycling scavenges superoxide anion radicals. This enzyme exerts anti-inflammatory effects, stabilizes p53 and potentially other tumor suppressors (Lee et al. 2021). The other genes commonly upregulated between female transgenic mice and female LC patients were epiregulin, fibrinogen γ, connexin 30 (*GJB6*) and *CD177*. Conversely, the only repressed gene commonly regulated between mouse and human lung tumors codes for aminopeptidase N. Based on Kaplan–Meier survival plots, epiregulin is prognostic; its high expression is associated with poor outcome (Fig. 5f). Additionally, the comparison of male LC patients and male cRaf mice defined 3 genes commonly upregulated (Fig. 5d). *CD177,* i.e., a GPI-anchored cell surface glycoprotein functions in neutrophil transmigration and tumor-infiltrating regulatory T cells. In fact, *CD177* is considered to be a bona fide target for Treg specific immunotherapies in solid cancers (Kim et al. 2021b). The second gene codes for the glycoprotein ORM1 which is typically upregulated during inflammation and may be regarded as an early stage LC biomarker (Ye et al. 2020). The third gene codes for DLK1 (= delta-like non-canonical NOTCH ligand 1) which stimulates cell growth and was highly upregulated (23-fold). However, none of the genes were prognostic in Kaplan–Meier OS plots (Fig. 5f). Interestingly, in the Cox proportional hazard regression analysis the number of pack years did not influence outcome (Supplementary Table S10).

Furthermore, we identified 89 (25 up, 64 downregulated) miRNAs specifically regulated in female LC patients (Supplementary Table S9), while for males, none fulfilled the threshold criteria FC >|2| and adj.*p* < 0.05 when compared to non-smokers. Possibly the number of controls were underpowered to identify significant miRNAs in males. Figure 5e shows the overlapping DEMs between female LC and mice lung tumors. Here, the tumor suppressor miR-146b-5p (two to threefold) and miR-339-5p (three to fourfold) were commonly repressed but neither are of prognostic value in Kaplan–Meier survival plots (Fig. 5f).

#### Is there a link between tumor size and sex specific gene regulations in human LC?

As compared to males, the tumor burden among transgenic females was significantly increased (Fig. 1). We therefore searched for genes mechanistically linked to tumor growth and focused on genes coding for MAPK signaling, tumor suppressors and oncogenes as well as cell proliferation. We only considered DEGs and DEMs that were specifically regulated in male and female LC patients (Fig. 5a).

**Fig. 6:**
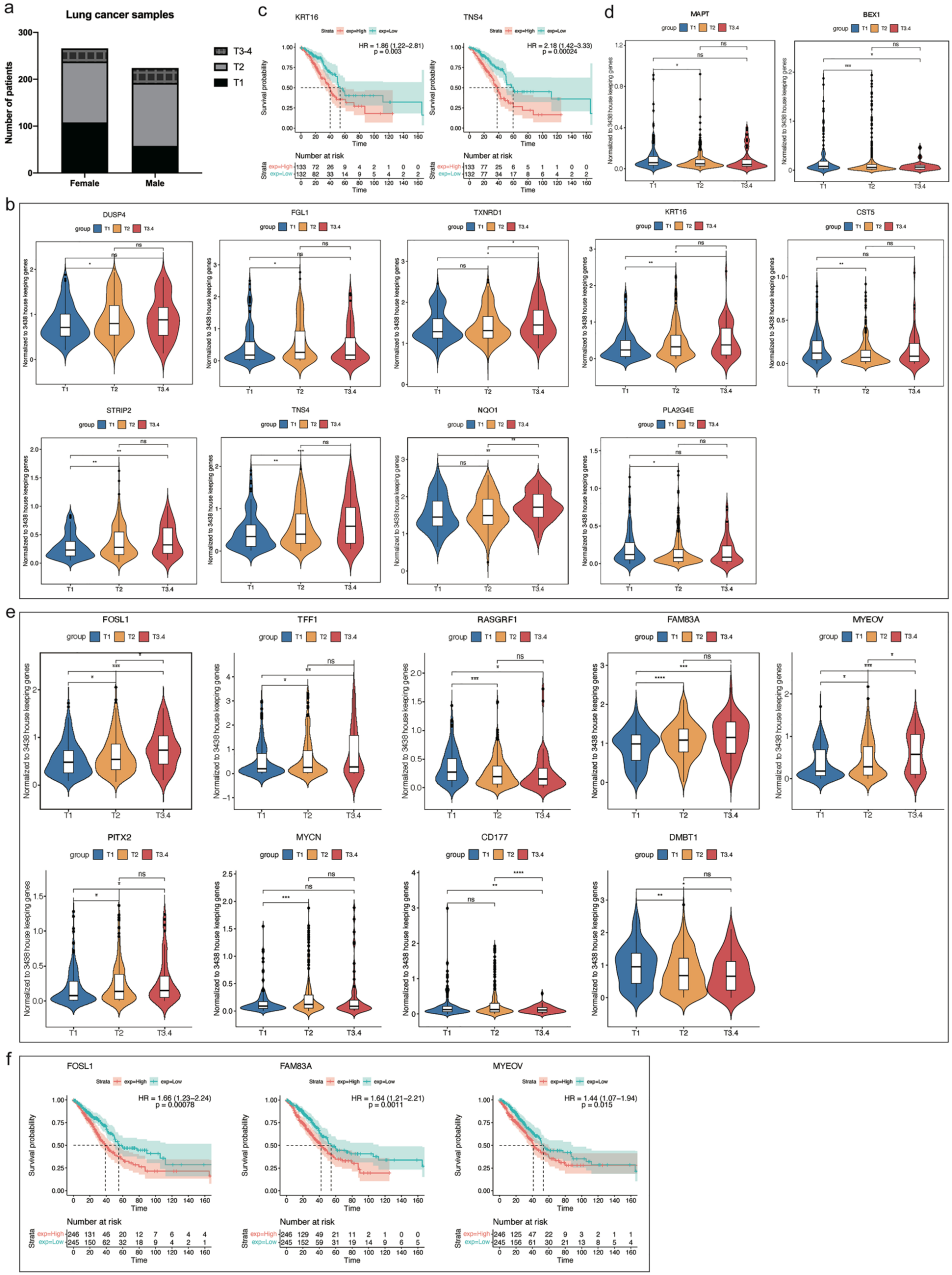
Identification of tumor size associated DEGs in LC patients. **a** The distribution of tumor size of LC patients. **b** Violin plots showing DEGs specifically associated with tumor size in female LC patients. **c** Kaplan–Meier survival plots of DEGs specifically associated with tumor size of female LC patients. **d** Violin plots of the DEGs specifically associated with tumor size of male LC patients. **e** Violin plots of the sex-independent DEGs, whose expression was associated with tumor size of LC patients. **f** Kaplan–Meier plots of the sex-independent DEGs. *DEG* differentially expressed gene

Depicted in Fig. 6a is the distribution of tumor sizes among 266 female and 224 male LC patients. The number of cases staged T1 and T2 are comparable among female LC patients, i.e., 108 and 130 cases while for males there are 58 and 134 cases. For obvious reasons the cases staged T3 (tumor size 5 to 7 cm) are relatively small, i.e., 28 and 32 cases among female and male LC patients. The majority are N0 (no lymph node invasion) and M0 (no evidence for metastasis). Due to the small number of cases staged T4, i.e., tumors > 7 cm, we combined the T3 and T4 cases in our analysis. Next, we considered DEGs uniquely regulated in female LC patients and determined their expression in relation to tumor size. We identified 9 genes whose increased expression was significantly associated with tumor size in female LC patients of which four code for MAPK signaling molecules (DUSP4, FGL1, TXNRD1, PLA2G4E), three for oncogenes (KRT16, STRIP2 and TNS4), tumor suppressor cystatin 5, and NQO1(Fig. 6b). Of these genes, four were regulated by the estrogen receptor (TXNRD1, TNS4, PLA2G4E and NQO1), and high expression of KRT16 and TNS4 is associated with poor survival in Kaplan–Meier survival plots (Fig. 6c). The results for male LC patients are given in Fig. 6d, and induced expression of MAPT and BEX1 was associated with tumor size; however, none were prognostic for OS.

Finally, we searched for sex independent DEGs whose expression was associated with tumor size (Fig. 6e). There are nine genes of which three code for MAPK signaling, four oncogenes and the remaining two code for the tumor suppressor DMBT1 and immune response (CD177). Note repressed expression of RASGRF1 and DMBT1 was associated with increased tumor size, and high expression of FOSL1, FAM83A and MYEOV was associated with poor survival (Fig. 6f).

Together, we identified 20 genes which were significantly associated with tumor size of which nine and two, respectively, are uniquely regulated in female (Fig. 6b) and male LC patients (Fig. 6d). Additionally, we identified nine genes significantly associated to tumor size which were independent of sex (Fig. 6e). Collectively, we observed a sex disproportional regulation of genes mechanistically linked to tumor growth (Supplementary Table S9), and we confirmed the cRaf-dependent regulations of CD177 and NQO1 in females LC patients.

**Fig. 7:**
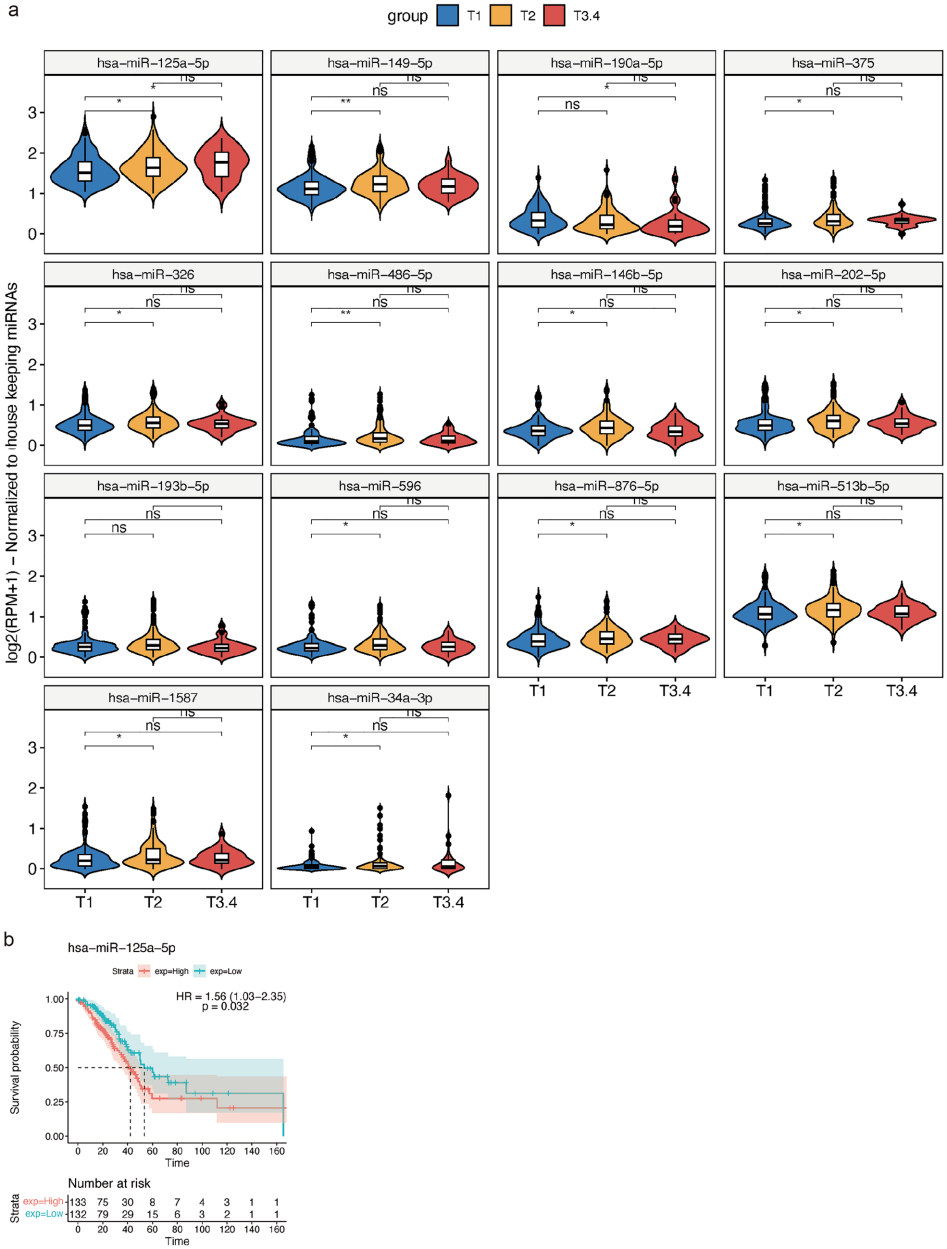
Identification of tumor size associated DEMs in LC patients. **a** Violin plots of 14 DEMs whose expression was associated with female LC tumor size. **b** Kaplan–Meier plot for miR-125a-5p. High expression is associated with worse outcome. *DEM* differentially expressed miRNA

We took the same approach to identify miRNAs associated with tumor size, and this defined 14 miRNAs, of which one code for oncomirs (upregulated), and 12 for tumor suppressors (one up, 11 downregulated) (Fig. 7a). All these miRNAs were uniquely regulated in female LC patients and their association with tumor size is mechanistically plausible (Supplementary Table S9). However and except for miR-125a-5p, none are prognostic in Kaplan–Meier survival plots (Fig. 7b).

## Discussion

Our study aimed at investigating the tumor promoting effect of tobacco smoke on tumor growth in a cRaf transgenic disease model, and we report the complex interplay of cRaf, tobacco smoke and sex hormones in the regulation of tumor suppressors, oncogenes and oncomirs. Additionally, we confirmed clinical relevance in a large cohort of LC patients.

### Tobacco smoke desynchronized the circadian clock

We found tobacco smoke exposure to significantly desynchronize the circadian rhythm by regulating Npas2 and Arntl. These bHLH-PAS TFs form heterodimeric protein complexes and bind to cognate recognition sites in the promoters of targeted genes. Depending on the dimerization partner the expression of either defense genes such as *CYP1A1* and *CYP1B1* or circadian clock genes is augmented (Fribourgh and Partch 2017). We identified a regulatory loop whereby tobacco smoke exposure of transgenic mice induced expression of Per2/3 which in turn repressed Arntl/Bmal1-dependent expression of circadian clock target genes. In fact, Arntl heterodimerizes with Clock to control circadian gene expression (Fig. 2h). Although *Clock* itself was unchanged, tobacco smoke exposure caused the near silencing of the *Clock* paralogue *Npas2* to about 10% of sham exposed controls. In fact, genetic studies revealed Npas2 to substitute Clock function as shown for Clock ko mice (DeBruyne et al. 2007), and our findings led us to propose a negative feedback loop whereby tobacco smoke exposure activated Per2/3 to repress Npas2 and Arntl activity. Furthermore, we found Arnt2 > threefold induced in tobacco smoke exposed transgenic females (Supplementary Table S3), and this TF in conjunction with the Ahr forms a heterodimeric protein complex to stimulate expression of defense genes such as *Cyp1a1* and *Cyp1b1* which were > 10- and > 24-fold induced.

There is strong evidence for Arntl/Bmal1 deficiency to result in hyperactivity of mTOR (Khapre et al. 2014). Moreover, in a recent review the importance of p53 dysfunction and mTOR pathway hyperactivity in human cancers has been highlighted (Cui et al. 2021). Tobacco smoke exposure caused marked repression of *Arntl/Bmal1* in both wild type and cRaf transgenic animals (Supplementary Table S3) and given that tobacco-carcinogens activate mTOR signaling (Mendoza et al. 2011; Memmott and Dennis 2010), we hypothesized mTOR hyperactivity to be the cause of an increased lung tumor burden (Fig. 1). In fact, we observed several molecules regulated in the Ras-Erk and mTOR pathway and next to cRaf itself, we measured > 27-fold induced expression of the EGFR ligand epiregulin. Similarly, the sixfold induced alpha fetoprotein expression likely contributed to PTEN inhibition and, therefore, stimulated PI3K/Akt/mTOR signaling as shown for human hepatoma cell lines (Li et al. 2011). A further example relates to the *Nqo1* upregulation, and this protein inhibits the phosphatase PP2A at the intersection of the PI3k/Akt and MAPK pathways (Li et al. 2015b). Additionally, mTOR responsive downstream targets such as *Cdk1* and *Mmp9* were regulated in tobacco smoke exposed animals. Collectively, we observed regulatory loops running in parallel, i.e., the circadian clock and the Ahr-dependent xenobiotic defense loop, and we propose tobacco smoke induced repression of *Arntl/Bmal1* to stimulate mTOR signaling.

We also identified key circadian genes as targets of sex hormone receptors, and propose a circuitry which consisted of *Esr1*, miR-22-3p and circadian genes. The data infer a complex interplay between tobacco smoke, circadian rhythm and sex. Recently, a study reported a sex-dependent association between circadian genes and prognosis of colon cancer patients, and women with high tumor Cry1 expression had a worse outcome (Hasakova et al. 2018).

Finally, we observed a sex disproportional effect of tobacco smoke among female and male LC patients. Notwithstanding, we were unable to confirm the regulation of clock genes in LC patients as seen in cRaf transgenic mice. While at its infancy, there is growing evidence for the circadian clock to function as an important regulator of immunity in cancer (Zhang et al. 2021).

### Immune response and regulation of oncogenes in tobacco smoke exposed animals

With tobacco smoke exposed transgenic mice, we identified 19 macrophage-related marker genes (Supplementary Table S5) of which 9 code for TAMs. These convey immunosuppression and are associated with poor prognosis of cancer patients (Petty and Yang 2017; Ma et al. 2022). We identified upregulated macrophage receptor with collagenous structure (*Marco*) in tobacco smoke exposed animals, and its induced expression is typically observed in immunosuppressive TAMs. In fact, the macrophages in tumor cell nests co-express PD-L1 and MARCO (Fleur et al. 2018), and a recent study demonstrated MARCO-expressing TAMs to suppress T and NK cell activity. Conversely, blocking MARCO restored their activity towards tumor cells (Fleur et al. 2021). This profiles MARCO as a therapeutic target to remodel the immune-suppressive microenvironment in LC. Indeed, the complex role of TAMs in enhancing tumorigenesis, metastasis and chemo-resistance has been the subject of several reviews (Xu et al. 2020; Condeelis and Pollard 2006). In addition, we identified spondin 2 (Spon2) as > threefold repressed in tobacco smoke exposed animals. This extracellular matrix protein is of critical importance for M1-like macrophages and lymphocyte recruitment (Zhang et al. 2018), and can be regarded as an anti-inflammatory response. Moreover, chemokine ligand 3 was significantly repressed following tobacco smoke exposure of mice. Together, we noted repression of several inflammatory cytokine enriched TAM marker genes in tobacco smoke exposed cRaf transgenic mice (Ma et al. 2022). Furthermore, we found *Cd177*, *Ogt*, *Dusp10* and *Zbtb16* upregulated, and these genes are regulators of leukocyte activation. Conversely, *Nfil3* and *Adm* were repressed, which function as regulators of dendritic cell activation (Kashiwada et al. 2011a; Rullé et al. 2012).

Importantly, we identified 14 oncogenes (13 up, one downregulated) and 11 tumor suppressors (eight up, three downregulated) in tobacco smoke exposed mice. Their regulation provide a rationale for the tumor promoting effects of tobacco smoke. Specifically, actinin-α was nearly fourfold upregulated in tobacco exposed mice and this cytoskeleton protein stimulates cell proliferation by inhibiting Hippo signaling as shown in hepatocellular carcinoma (Chen et al. 2021). Similarly, tobacco smoke caused induced expression of the MYC-interacting transcriptional modulator Cbp/P300. This coactivator interacts with histone deacetylase 1 and functions as a molecular switch of cytokine-induced cell proliferation (Chou et al. 2012). We already described the tumor promoting effect of cathepsin K (Yang et al. 2020), and of lysine demethylase Kdm2a in LC which stimulates Erk1/Erk2 signaling through epigenetic silencing of DUSP3 (Wagner et al. 2013). Once again, tobacco smoke exposure caused their induced expression and similar findings were obtained for the cell-surface protein *Tspan4* which promotes cell proliferation and invasion in gastric cancers (Deng et al. 2021). Other examples of tobacco smoke induced tumor promoters are the microtubule associated monooxygenase, i.e., calponin and LIM domain containing 2 that functions in cytoskeletal dynamics, migration and EMT in LC (Zhou et al. 2020). Additionally, we observed upregulation of *Ccnjl*, *Nr1d2* and *Sox13*, which stimulate EMT, cell migration and invasion (Papagiannakopoulos et al. 2016; Du et al. 2020; Pacheco-Pinedo and Morrisey 2011). Moreover, tobacco smoke repressed the expression of the tumor suppressors *Arntl* and *Npas2* in females*,* and their repression promoted cell proliferation in various cancers (Supplementary Tables S3, S6) (Zhang et al. 2018; Papagiannakopoulos et al. 2016; Hoffman et al. 2008).

Although tobacco smoke caused marked repression of several tumor suppressors, it also elicited upregulation of tumor suppressors in males. These code for apoptosis (*Acer2*, *Zbtb16*), inhibit cell migration and invasion (*Per2&3*, *Hlf*) and block Erk signaling (*Dusp10*) (Wang et al. 2013, 2017a; Chen et al. 2020; Jiménez-Martínez et al. 2019; Xiang et al. 2018; Tang et al. 2018).

Furthermore, tobacco smoke influenced expression of 4 oncomirs (4 repressed) and 20 tumor suppressors (5 up, 15 downregulated). Of the 20 tumor suppressors, 12 were female specific (1 up, 11 downregulated) and 8 were male specific (4 up, 4 downregulated). For instance, let-7d-5p, let-7f-5p and let-7i-5p were uniquely repressed in females and provide a molecular rationale for the higher tumor burden among females (Fig. 1). Additionally, tumor suppressors miR-142-5p, miR-146b-5p, miR-16-5p, miR-200b-5p, miR-22-3p, miR-30c-2-3p, miR-335-5p and miR-339-5p were repressed in females only, and these function in MAPK signaling, cell proliferation, EMT, cell migration and invasion, angiogenesis and apoptosis (Wang et al. 2017c, 2020; Li et al. 2017, 2018; Chen et al. 2019; Yang et al. 2021; Jin et al. 2020).

### cRaf—tobacco smoke interactions

Finally, we evaluated the combined effects of tobacco smoke and cRaf on the pulmonary genome, and identified 33 DEGs and 13 DEMs as uniquely regulated by the combined activity of these two effectors (Fig. 4e). Of the 33 DEGs, five (upregulated) code for oncogenes and five (two up, three downregulated) for tumor suppressors.

A remarkable finding relates to an induced expression of *Apoc1*. This apolipoprotein tags lipid-associated TAMs, regulates macrophage polarization and promotes tumor metastasis in renal cancers (Ren et al. 2022). Likewise, induced expression of the glycoprotein Nmb is a marker gene of scar-associated macrophages (Ma et al. 2022). Additionally, piwi-like RNA-mediated gene silencing 4 and protein tyrosine phosphatase 2 promote cell migration and EMT in LC and breast cancer (Sengelaub et al. 2016; Wang et al. 2016), and were significantly upregulated in transgenic mice following tobacco smoke exposure. Moreover, with cRaf males, we observed repression of thyroid hormone responsive. Typically, the coded protein inhibits cell proliferation, migration and invasion in HCC (Hu et al. 2021). However, its repression likely contributed to tumor growth. Similarly, *S100a8* and *Htra3* were repressed. S100a8 hallmarks inflammatory TAMs (Ma et al. 2022), and Htra3 function as a tumor suppressor by stimulating apoptosis in LC (Wenta et al. 2019). Lastly, Rap1 GTPase-activating protein and Arnt2 were upregulated, and these inhibit cell migration, invasion (Tsygankova et al. 2013), proliferation, and promote apoptosis (Yang et al. 2015).

Among the 13 DEMs, nine function as tumor suppressors and we observed sex-dependent regulations. For instance, let-7c-5p, miR-150-5p inhibit cell metastasis, and induce apoptosis (Liu et al. 2022; Dai et al. 2019), and these miRNAs were specifically repressed in females. Conversely, miR-125-3p, miR-191-5p, miR-200c-3p, miR-30a-5p and miR-361-5p were specifically upregulated in males, and these tumor suppressors were reported to inhibit cell proliferation, migration and invasion in LC (Li et al. 2015a; Quan et al. 2019; Byun et al. 2019; Hou et al. 2017; Chen et al. 2018). Furthermore, miR-193a-5p suppresses LC metastasis by targeting ERBB4/PIK3R3/mTOR/S6K2 (Li et al. 2018), and this tumor suppressor was upregulated in males but downregulated in females.

**Fig. 8:**
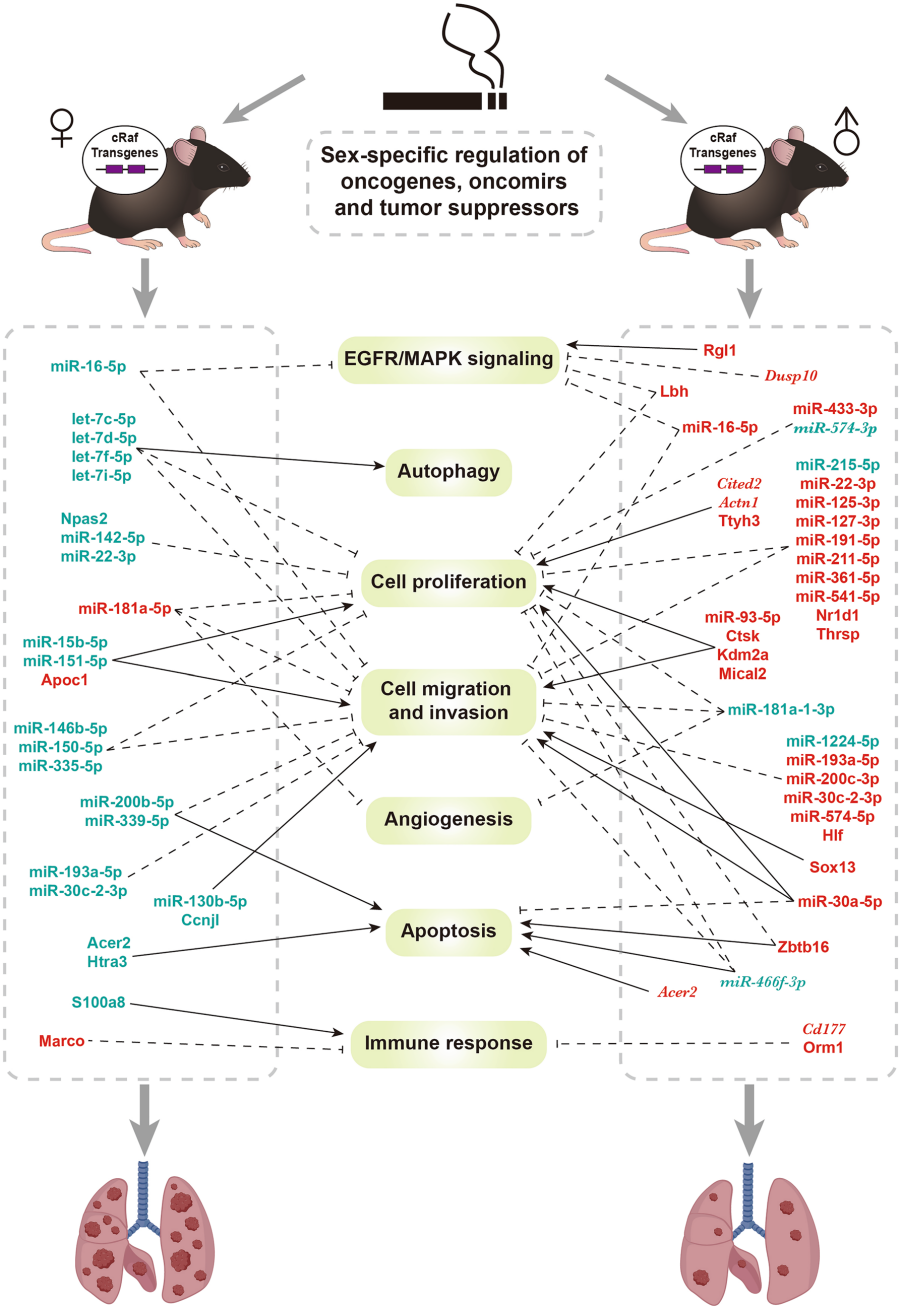
Sex-specific regulation of oncogenes, oncomirs, and tumor suppressors in tobacco smoke exposed mice. Depicted are sex-specific regulations of tobacco smoke exposed cRaf transgenic mice. Up and downregulated genes and miRNAs were marked in red and turquoise. Genes and miRNAs marked in italic were only regulated in tobacco smoke exposed WT animals. The function of the genes is highlighted by arrows (activation) or dashed lines (inhibition). Tobacco smoke promotes tumor growth irrespective of sex. However, females are more vulnerable as evidenced by tumor size and multiplicity. We propose a molecular circuitry underlying gender specific responses to tobacco smoke and show male transgenic mice to be partly protected by the specific upregulation of 20 tumor suppressors and genes coding for programmed cell death. Notwithstanding, there are 10 and 2 genes, respectively, upregulated in tobacco smoke exposed transgenic males that support tumor growth and immune evasion. Conversely, in tobacco smoke exposed females 18 tumor suppressors were downregulated thus influencing, EGFR signaling, autophagy cell proliferation cell migration and program cell death. Furthermore, *Apoc1* and *Marco* were upregulated to stimulate cell proliferation and immune evasion. In fact miR-181a-5p is the only tumor suppressor specifically upregulated in tobacco smoke exposed females. *WT* wild type

Figure 8 depicts a summary of the findings. We highlight the tumor promoting effects of tobacco smoke as it relates to EGFR/MAPK signaling, cell migration and invasion, cell proliferation, immune response, programmed cell death and autophagy. The results document the pleiotropic effects of tobacco smoke in stimulating sex-dependent LC growth with females presenting higher tumor burden.

## Conclusion

Our study provides insight into the genomic landscape of the sex-related difference of LC growth, and we report the molecular wiring of lung tumors in tobacco smoke exposed cRaf transgenic animals. We identified genes associated with tumor growth and validated the findings in a large cohort of LC patients.

### Supplementary Information

Below is the link to the electronic supplementary material.

Supplementary file1 (DOCX 1510 KB)

Supplementary file2 (DOCX 782 KB)

Supplementary file3 (DOCX 17 KB)

Supplementary file4 (XLSX 12 KB)

Supplementary file5 (XLSX 45 KB)

Supplementary file6 (XLSX 121 KB)

Supplementary file7 (XLSX 26 KB)

Supplementary file8 (XLSX 32 KB)

Supplementary file9 (XLSX 12 KB)

Supplementary file10 (XLSX 11 KB)

Supplementary file11 (XLSX 122 KB)

Supplementary file12 (XLSX 9 KB)

## Data Availability

The data to support the findings of this study are available from the authors upon reasonable request.
