# Supplementary material for: Sex differences in the tumor promoting effects of tobacco smoke in a cRaf transgenic lung cancer disease model
Source: Arch Toxicol. 2024 Jan 21;98(3):957–83. doi: 10.1007/s00204-023-03671-5 (PMC10861769; doi:10.1007/s00204-023-03671-5)
Supplement: Supplementary file 1 — Supplementary file1 (DOCX 1510 KB) [file 204_2023_3671_MOESM1_ESM.docx]

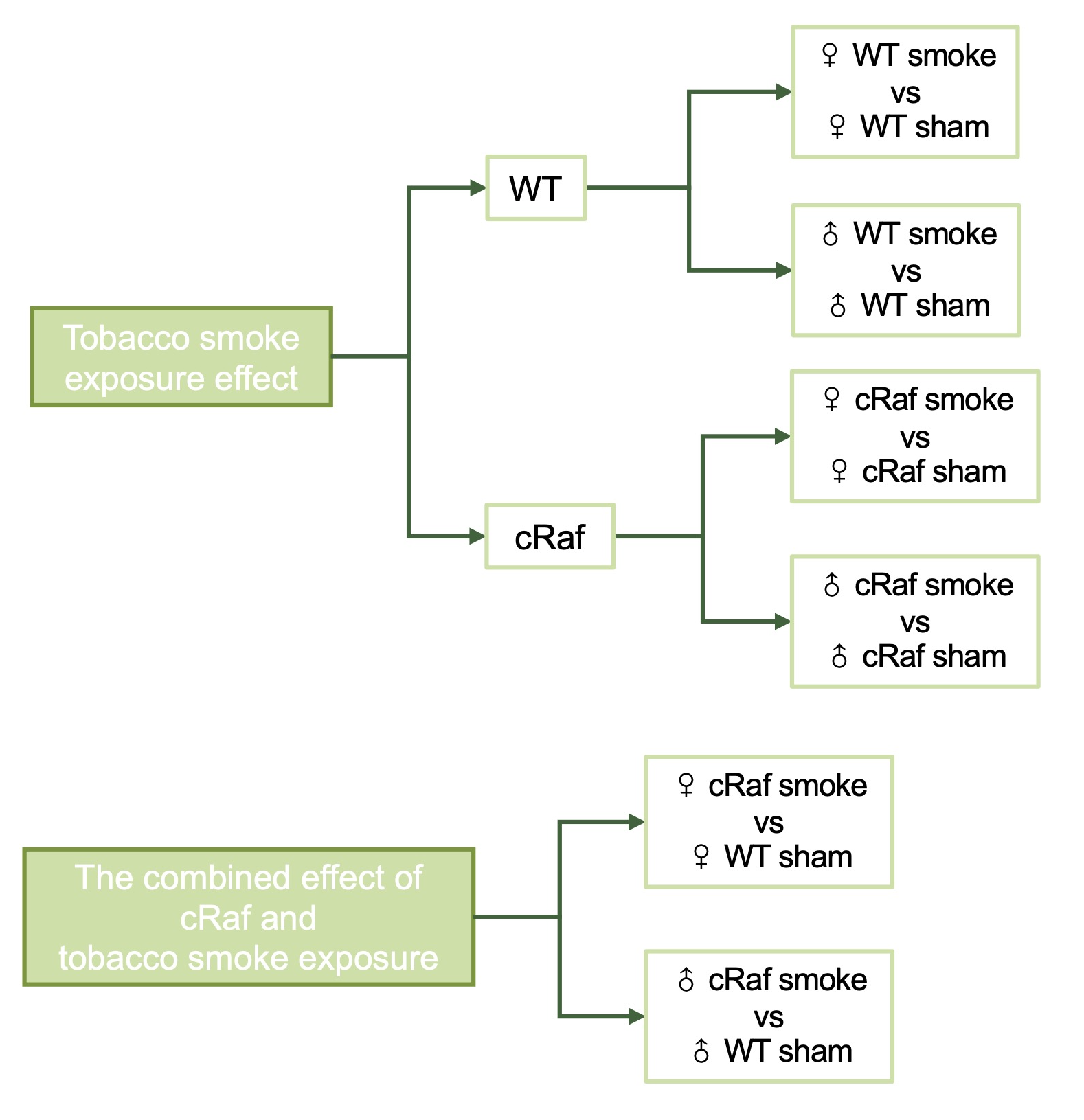


Supplementary Figure S1. The various comparisons of the study.


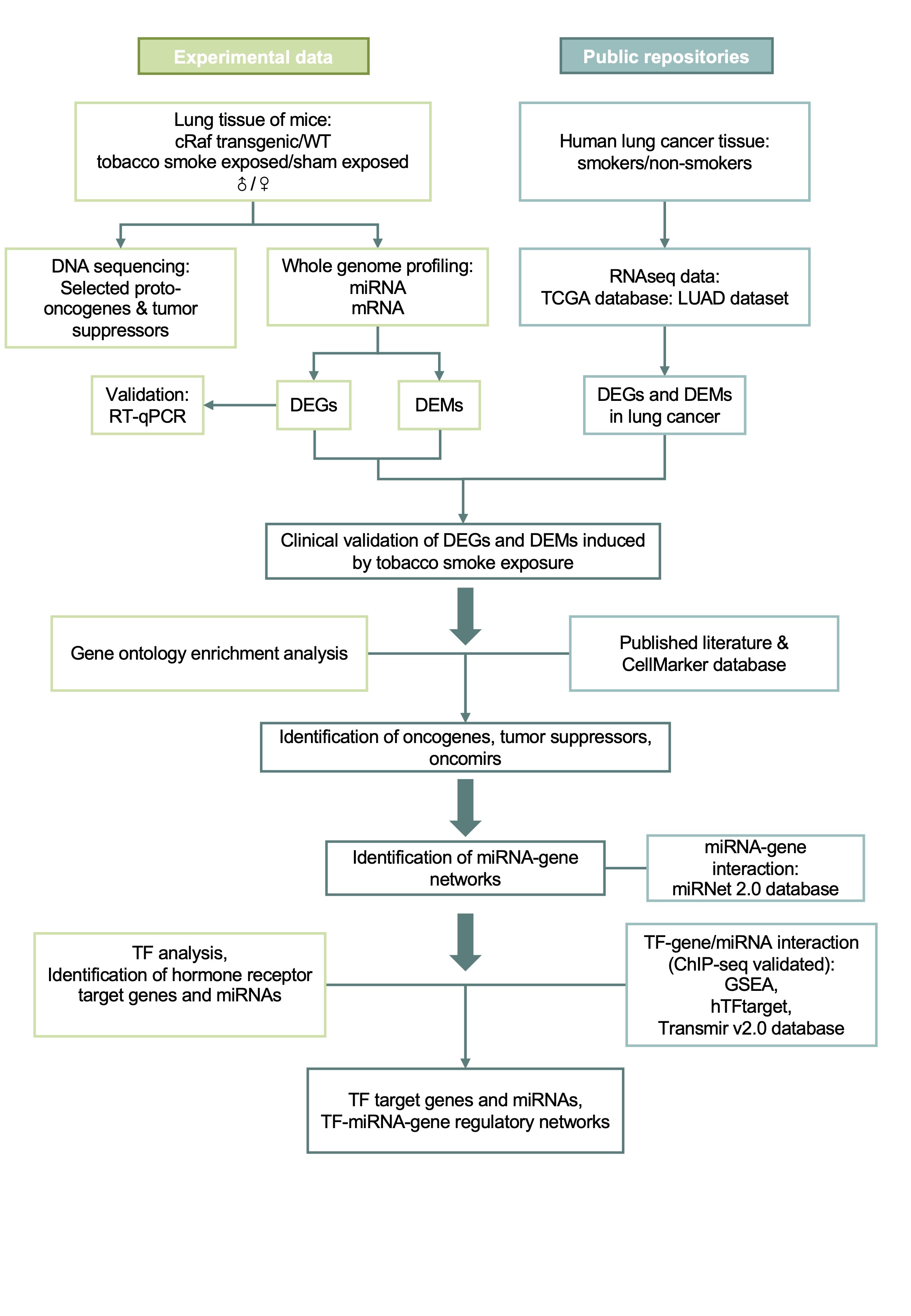


Supplementary Figure S2: The research strategy of the study.


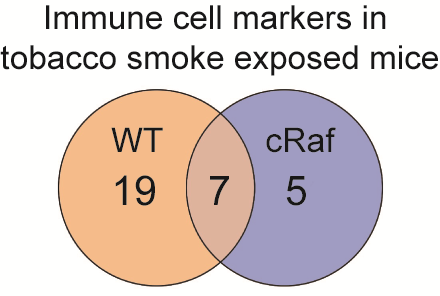


Supplementary Figure S3: Venn diagram of immune cell marker genes in WT and cRaf animals.
