# Supplementary material for: Sex differences in the tumor promoting effects of tobacco smoke in a cRaf transgenic lung cancer disease model
Source: Arch Toxicol. 2024 Jan 21;98(3):957–83. doi: 10.1007/s00204-023-03671-5 (PMC10861769; doi:10.1007/s00204-023-03671-5)
Supplement: Supplementary file 2 — Supplementary file2 (DOCX 782 KB) [file 204_2023_3671_MOESM2_ESM.docx]

Sequence analysis of genes associated with lung tumors

Genomic DNA isolation and PCR amplification

We isolated genomic DNA from lung tissues of cRaf transgenic animals (Figure 1), and amplified all gene fragments for sequence analysis, which were within 500 bp. (see Figure 2 for the amplification of exon 2 of *Lmyc1*), and directly performed sequencing. Together, we analyzed 4 lung cancer (LC) associated oncogenes (*Lmyc1, Kras*) and tumor suppressors (*Trp53, Tslc1*) in tobacco smoke and sham cRaf animals, however did not detect any sequence alterations. We show the details below.

Figure 1: Genomic DNA isolated from mouse study samples. Genomic DNA (5 µl) from each sample was analyzed on 1% ethidium bromide gel using as size marker (M) 1 kb-plus ladder, and known 200 ng and 400 ng lambda DNA concentrations.

Figure 2: PCR amplification of *Lmyc1* (exon 2C) from isolated genomic DNA in study samples. PCR product (5µl) from each study sample analyzed on 1% ethidium bromide gel. 1 kb-plus ladder (M), arrows point to amplified PCR fragments.

Mutation detection in Lmyc1, Trp53, Tslc1, Kras

We sequenced amplified PCR fragments for *Lmyc1, Kras*, *Trp53*, and *Tslc1*, (Figure 3a-S3d).

Figure 3a: Example of a sequence electropherogram for the analysis of *Lmyc1*.

Figure 3b: Example of a sequence electropherogram for the analysis of *Tp53.*

Figure 3c: Example of a sequence electropherogram for the analysis of *Tslc1.*

Figure 3d: Example of a sequence electropherogram for the analysis of *Kras.*

We performed fluorescent Sanger sequencing with the Big Dye Terminator v 3.1 sequencing kit according to the manufacturers recommendation (ThermoFischer, Germany) on a capillary electrophoresis Genetic Analyzer 3100 (Applied Biosystems). We compared sequence with the SeqScape software.

#### Lmyc1

We determined whether *Lmyc1* contained sequence alterations in the study sample (Figure 4a-4b). *Lmyc1* gene is located on chromosome 4 and consists of two exons: Exon 1 with 920 bp and exon 2 with 2581 bp. Lmyc1 consists of 368 amino acids and contains a basic helix-loop-helix motif (aa 286-338). Analysis of the sequences encoding the protein (four PCR fragments, 1823 bp) in tobacco smoke exposed cRaf animals (16 males, 16 females), cRaf sham animals (16 males, 16 females) did not reveal any mutations.

Figure 4a: Schematic diagram of *Lmyc1* genomic structure, PCR primer sequence and conditions.

Figure 4b: Detection of mutations in *Lmyc1*, showing examples of PCR products and a sequence electropherogram.

#### Trp53

*Trp53* is located on chromosome 11 and consists of 11 exons. The sequences encoding the 390 amino acid-protein span 10 exons, and the sequences encoding the DNA binding domain (aa 99-289) are located in exons 4-8. To analyze the coding sequences, we amplified eight fragments (exons 3 and 4 or exons 8 and 9 were combined into 1 fragment) (Figure 5a-5b). We sequenced samples for tobacco smoked exposed cRaf animals (16 males, 16 females) and did not identify sequence alterations.

Figure 5a: Schematic diagram of Trp53 genomic structure, PCR primer sequence and conditions.

Figure 5b: Detection of mutations in *Trp53*, showing examples of PCR products and a sequence electropherogram.

#### Tslc1

The *Tslc1* gene maps to chromosome 9 and is composed of 10 exons. The exon-intron junctions are highly conserved between human and mouse and the mouse cDNA contains a single open reading frame of 1335 bp, which encodes a protein of 445 amino acids. The mouse Tslc1 amino acid sequence display 98% identity with that of human TSLC1.

We amplified the coding sequence of *Tslc1*, which spans 10 exons, resulting in 10 PCR fragments with an average length of 387 bp (312-487 bp), and a total of 3867 bp for each sample (Figure 6a-6b). We sequenced exons 1-10 of tobacco smoke exposed cRaf animals (16 females, 4 males) and did not identify sequence alterations in this tumor suppressor.

Figure 6a: Schematic diagram of *Tslc1* genomic structure, PCR primer sequence and conditions.

Figure 6b: Detection of mutations in *Tslc1*, showing examples of PCR products and a sequence electropherogram.

#### Kras

The *Kras* gene is located on chromosome 6 and consists of four exons: exon 1 (111 bp), exon 2 (179 bp), exon 3 (160 bp) and exon 4 (120 bp) and codes for 189 amino acids. We specifically analyzed hotspot mutations in codon 12 and 13 in exon 1, as well as codon 61 in exon 2 (Figure 7a-7b). For exon 1, we sequenced 16 tobacco smoke exposed cRaf females, and for exon 2, we sequenced 16 female and four male tobacco smoke exposed cRaf animals. Sequence analysis did not reveal any mutations.

Figure 7a: Schematic diagram of *Kras* genomic structure, PCR primer sequence and conditions.

Figure 7b: Detection of mutations in *Kras*, showing examples of PCR products and a sequence electropherogram.
