# Supplementary material for: Sex differences in the tumor promoting effects of tobacco smoke in a cRaf transgenic lung cancer disease model
Source: Arch Toxicol. 2024 Jan 21;98(3):957–83. doi: 10.1007/s00204-023-03671-5 (PMC10861769; doi:10.1007/s00204-023-03671-5)
Supplement: Supplementary file 3 — Supplementary file3 (DOCX 17 KB) [file 204_2023_3671_MOESM3_ESM.docx]

Table S1: Experimental groups

| Mice strain | Cigarette type | TPM concentration (μg/L) | Inhalation period (months) | Sex | Number of mice – miRNA expression | | Number of mice –gene expression | Number of mice-histopathology |
| --- | --- | --- | --- | --- | --- | --- | --- | --- |
|  |  |  |  |  | Agilent | Affymetrix |  |  |
| cRaf-transgenic | -(sham) | 0 | 3 | Male | 6 | 6 | 16 | 8 |
|  |  |  |  | Female | 6 | 6 | 16 | 8 |
|  | 2R4F | 150 | 3 | Male | 6 | 6 | 16 | 8 |
|  |  |  |  | Female | 6 | 6 | 16 | 8 |
| Non-transgenic | -(sham) | 0 | 3 | Male | 6 | 3 | 16 | 8 |
|  |  |  |  | Female | 6 | 3 | 16 | 8 |
|  | 2R4F | 150 | 3 | Male | 6 | 0 | 16 | 8 |
|  |  |  |  | Female | 6 | 0 | 16 | 8 |

TPM: total paticular matter
